# Synthesis, Characterization and Biological Studies of Some Novel Thieno[2,3-d]pyrimidines

**DOI:** 10.3390/molecules15063932

**Published:** 2010-06-01

**Authors:** Khulud M. Al-Taisan, Hassan M. A. Al-Hazimi, Shar S. Al-Shihry

**Affiliations:** 1 Chemistry Department, College of Science, King Faisal University, P.O. Box 400, Hofuf 31982, Saudi Arabia; 2 Chemistry Department, College of Science, King Saud University, P.O. Box 2455, Riyadh 11451, Saudi Arabia

**Keywords:** thienopyridines, triazolothienopyrimidines, antibacterial activity, cytotoxic activity

## Abstract

Several 2-unsubstituted thieno[2,3-d]pyrimidines have been prepared from 2-aminothiophene-3-carboxylic acid esters and their carbonitrile analogs. Some triazolo-thienopyrimidine and 2-thioxothienopyrimidine representatives have also been synthesized using thermal and microwave (MW) irradiation techniques. Structures of the prepared compounds were elucidated on the basis of IR, NMR, 2D NMR and mass spectral data. The biological activity of some selected synthesized compounds was also examined.

## 1. Introduction

**Figure 1:**
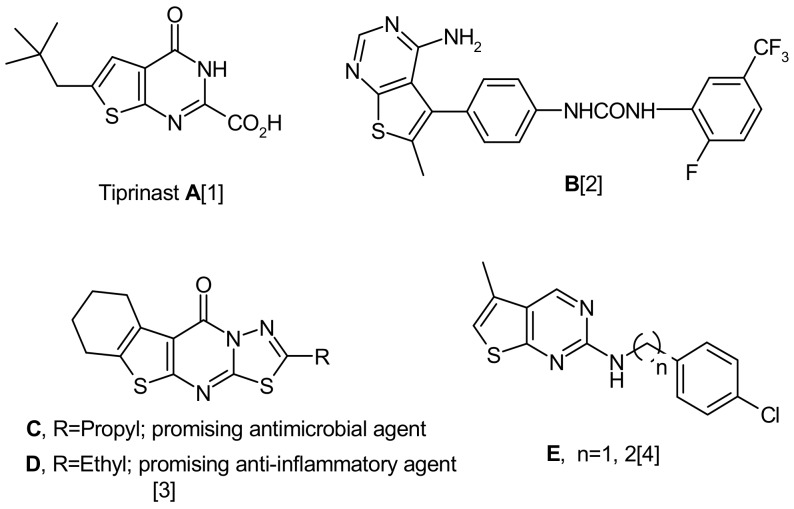
Some biologically active thienopyrimidines.

Heterocycles containing the thienopyrimidine moiety (Figure 1) are of interest because of their interesting pharmacological and biological activities [1,2,3,4,5,6]. Thus, over the last two decades many thienopyrimidines have been found to exhibit a variety of pronounced activities, for example, as antiinflammatory [3,7], antimicrobial [3,8], antiviral [9] and analgesic [7,10] agents. Some thienopyrimidine derivatives showed good antitumor activity [11], while compounds with the general structure designated by E (Figure 1) showed potent and specific cytotoxicity against several leukemia cell lines [4]. Motivated by the aforementioned biological and pharmacological importance of the title compounds, and as continuation with our previous work on thienopyrimidines [12,13], we report herein the synthesis of some new heterocycles incorporating a thienopyrimidine moiety. Representative compounds among the synthesized thienopyrimidines were tested and evaluated as antibacterial agents and for cytotoxicity against some cancer cell lines.

## 2. Results and Discussion

### 2.1. Chemistry

**Scheme 1:**
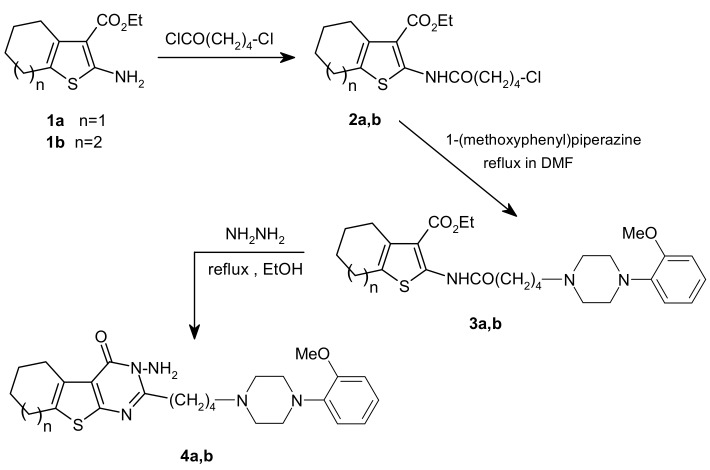
Synthesis of compound **2–4**.

**Scheme 2:**
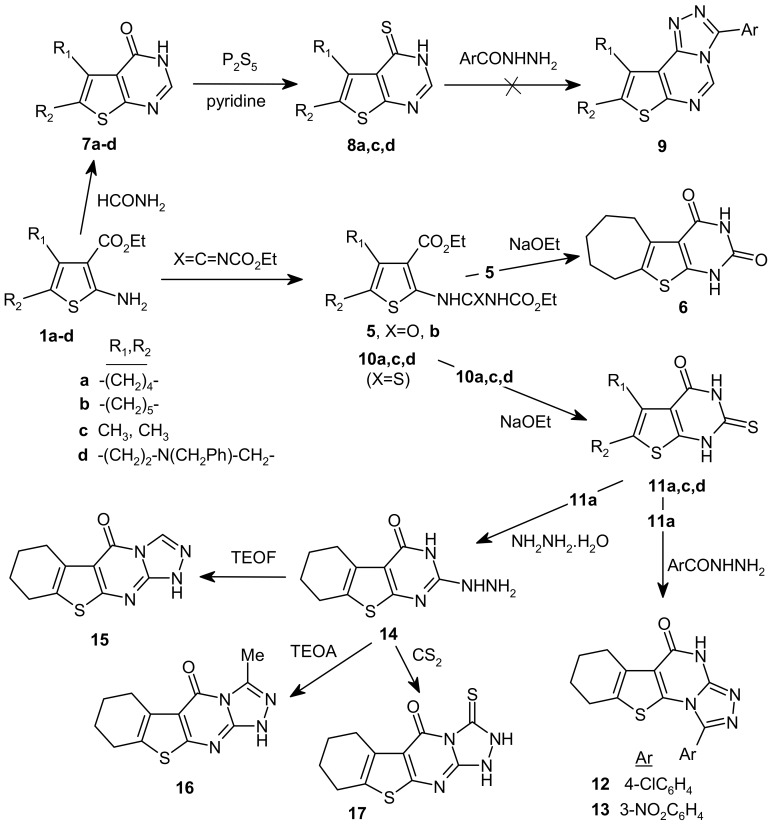
Synthesis of compounds **5–17**.

**Scheme 3:**
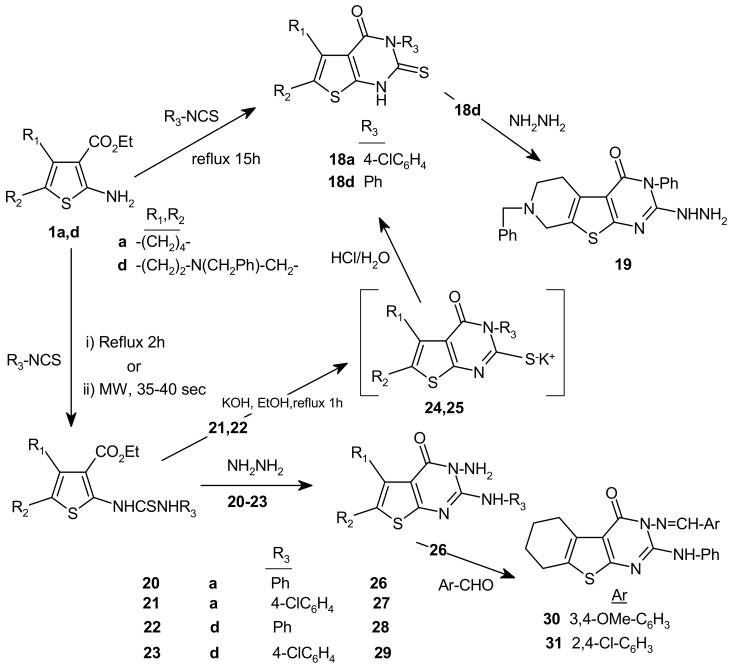
Synthesis of compounds **18–31**.

**Scheme 4:**
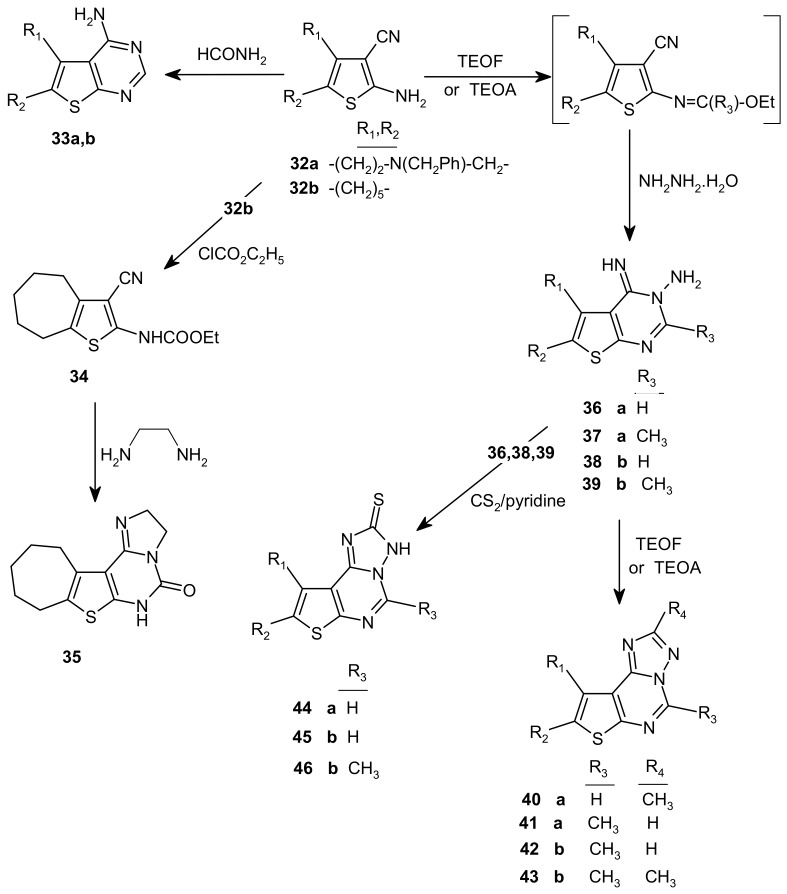
Synthesis of compounds **32–46**.

The synthetic pathways depicted in Scheme 1, Scheme 2, Scheme 3 and Scheme 4 outline the chemistry of the present study. Thus, the 2-amino-3-thiophene carboxylic ester starting materials **1a-d** and **32a,b** are easily prepared following the well established procedure reported in the literature [14,15]. Treatment of **1a,b** with ClCO(CH_2_)_4_Cl gave the corresponding amides **2a,b**, which in turn gave compounds **3a,b** upon refluxing in DMF with 1-(methoxyphenyl)piperazine in the presence of potassium carbonate (Scheme 1). The IR spectra of **2a,b** and **3a,b** showed two peaks in the 1,675–1,678 cm^-1^ and 1,658–1,662 cm^-1^ range corresponding to ester and amide carbonyl absorptions, respectively. The structure of the unpreviously unknown **2a,b** and **3a,b** were unambiguously assigned on the basis of their NMR and MS data (see Experimental). Reaction of **3a,b** with hydrazine in hot methanol led to cyclization to the corresponding thienopyrimidines in moderate yields. The IR spectra of **4a,b** exhibited a strong peak corresponding to the carbonyl group at 1,672–1,680 cm^-1^. The ^1^H-NMR spectrum of **4a** in CDCl_3_ showed a broad singlet at δ 4.96 (NH_2_), a methoxyl proton singlet and four aromatic proton resonances (Table 1;). This spectrum also showed the presence of the cyclohexane and piperazine ring methylene protons as well as the butyl moiety, as judged from the DEPT spectrum. The mass spectrum of **4a** showed a prominent molecular ion peak [M^+^] as the base peak and a fragmentation pattern consistent with its structure. The ^13^C-NMR, combined with the DEPT spectrum of **4a**, confirmed the existence of 25 carbons, including a benzene ring, 12 methylene carbons (10 signals, taking in consideration the equivalency of methylene protons in the piperazine ring), methoxyl and thienopyrimidine carbons. HMBC spectroscopy (Table 1;) were used to elucidate the structure and to establish the complete NMR assignments of **4a**. Starting from H-2′ the most shielded aromatic proton, showed two-bond correlations with C-1′ and C-3′, and showed three-bond correlations with C-4′ and C-6′. H-5′ and methoxyl protons showed three-bond correlations with C-1′. Further, H-5′ and C-a′ of the piperazine ring showed respectively two-bond and three-bond correlations to C-6′.

A two bond-correlation observed from a′-CH_2_ to C-a, established the assignments of the chemical shifts of the piperazine ring methylene groups. H-1ʺ and the amino protons showed two-bond and three-bond correlations with C-2, respectively. The three-bond correlation from a-CH_2_ to C-4ʺ assigned the chemical shifts of the butyl protons in compound **4a**. On the other hand, the 9-CH_2_ showed three-bond correlations with C-5 and C-7 and two-bond correlations with C-6 and C-10, while 12-CH_2_ exhibited three-bond correlation to C-6 and two-bond correlations to C-7 and C-11 carbons. These correlations unequivocally confirmed the assignments of all carbons of the cyclohexane and thiophene rings. The signal at δ_C_ 141.34 must correspond to C-8 since this carbon has no correlation with any protons in the HMBC spectrum of **4a**.The mass spectrum of **4b** exhibited a molecular ion peak at *m/z* 481. Its NMR spectral data are similar to the corresponding data of **4a** with an additional δ_H_ at 1.82 and δ_C_ at 23.11 for the methylene group.

In another pathway, thienopyrimidine dione **6** and the 2-unsubstituted-thienopyrimidines **7–9** have been synthesized as depicted in Scheme 2. The structures of **6–8** were elucidated on the basis of their various spectral data (see Experimental), including their mass spectral data which were consistent with the proposed structures. Compound **8** failed to give the condensation product **9**. None of the thienopyrimidines in Scheme 2 were previously prepared, with the exception of **7a,b** [13]. The ^1^H- NMR spectrum of **8a** revealed two singlets at δ 7.62 and δ 13.14, each integrating for one proton, corresponding to the protons at position 2 and for the NH, respectively. Compounds **11a,c,d** were synthesized from **10** upon treatment with sodium ethoxide. The IR spectra of **11a,c,d** are characterized by two bands in the 1,664–1,681 cm^-1^ and 3,157–3,427 cm^-1^ range due to the C=O and NH stretching frequencies, respectively. These spectra also showed a band in the 1,110–1,174 cm^-1^range, which corresponds to the C=S absorption in **11**. ^13^C-NMR spectra of the latter compounds revealed two signals at around δ 173 and 157 which were attributed to the C=S and C=O, respectively, in addition to the other carbon signals at the expected values. Two singlets at δ 4.43 and 4.24 in the ^1^H-NMR spectrum of **11d**, each integrating for two protons, correspond to the CH_2_ of the benzyl group and the CH_2_ at position 2 of the piperidine moiety in the molecule, respectively. This spectrum also displayed other two multiplet signals at δ 3.15 (CH_2_) and δ 3.39 (CH_2_) in addition to the signals of the protons for two NH groups at δ 11.40 (br s) and δ 12.50.

Compound **11a** reacted with hydrazine to give the corresponding hydrazino derivative **14** which in turn was transformed into the pyrazolothienopyrimidines **15–17** upon treatment with triethyl orthoformate, triethyl orthoacetate and carbon disulphide. A singlet at δ 9.01 in the ^1^H-NMR of **15** corresponds to the proton in the triazole ring, while a singlet at δ 2.98 in the similar spectrum of **16** corresponds to the methyl substituent in the latter heterocylic ring. Other than that, the data of both spectra were almost identical. Expected ^13^C-NMR chemical shifts for both compounds were observed (see Experimental).

**Figure 2:**
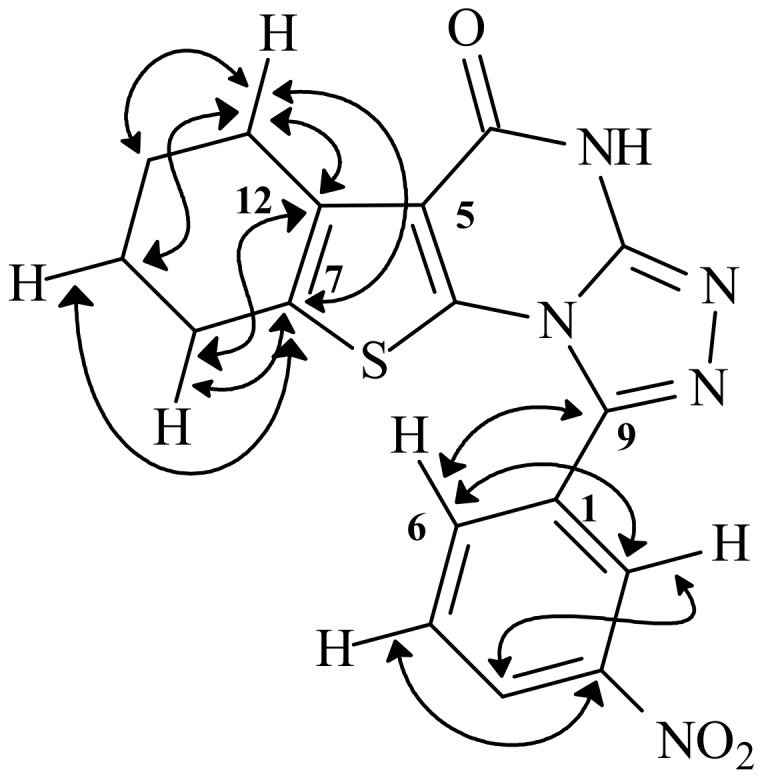
Selected HMBC spectrum of **13**.

The condensation products **12** and **13** were also obtained upon treatment of **11a** with some aromatic hydrazides, as elaborated in Scheme 2. Accordingly, cyclization of **11a** to the triazolo-derivatives **12** and **13** occured through the NH at position 1, as shown by the HMBC spectrum of **13** which indicated a two bond correlation of the hydrogen of NH at position 3 to the C-4, and in fact this would be the expected case where the latter NH is an amide. A literature survey revealed that these condensed thienopyrimidines **12–17** have not been previously prepared, and their NMR spectral data were in full agreement with the proposed structures. The assignment of the various carbons in the ^13^C-NMR spectra of **12–17** was accomplished with the aid of DEPT-135 and HETCOR experiments. The HMBC correlations of **13**, helpful in the assignment of chemical shifts, are shown in Figure 2.

The thioureido derivatives **20–23** were obtained in moderate yields upon reaction of **1a,d** with some aromatic isothiocyanates using either the classical heating method [12] or microwave irradiation [13], although the reaction was cleaner in the case of the latter method (Scheme 3). Thioureido compounds **21** and **22** were transformed to thienopyrimidine **18** through the corresponding pyridinium salts **24** and **25**, respectively. Compounds **18a,d** were obtained directly, although in low yields, on refluxing **1a,d** with the aromatic isothiocyanates for longer times. Condensation of **18d** with hydrazine hydrate gave the novel 2-hydrazino derivative **19**. Compounds **20–23** were also condensed with hydrazine hydrate to give the corresponding 3-aminothienopyrimidines **26–29**. The latter amines reacted smoothly with aromatic aldehydes, as exemplified by the reaction of **26** with two different aldehydes. Thienopyrimidines **28–31** have not been previously synthesized and their structures were firmly established on the basis of their NMR (δ_H,C_) and mass spectral data (see Experimental).

The starting 2-amino-3-cyanothiophene derivatives **32a,b** reacted with formamide to give the corresponding thienopyrimidine derivatives **33a,b** (Scheme 4). Structural elucidation of the latter was based on the various spectroscopic methods and comparison of the NMR data of **33b** with those given in the literature [13] for the same compound.

**Figure 3:**
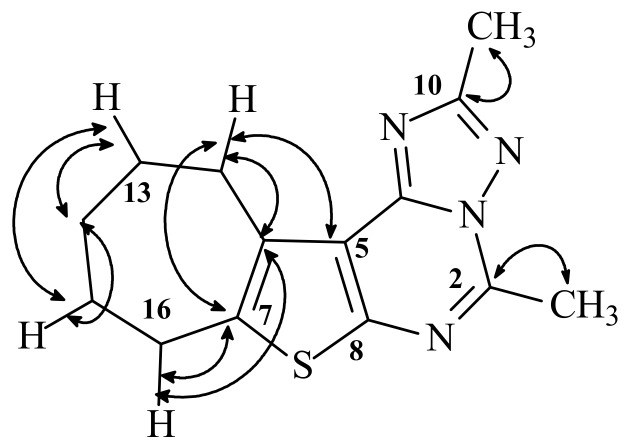
Selected HMBC spectrum of **43**.

Further, condensation of **32a,b** with TEOF and TEOA followed by hydrazine hydrate led to the formation of the novel thienopyrimidines **36-39**, whose structures were elucidated from their IR, NMR and MS spectral data (see Experimental). Compounds **40-43** were obtained in moderate yields upon the reaction of **37-39** with TEOF and TEOA. Reaction of **36**, **38** and **39** with carbon disulphide in pyridine gave the condensed thienopyrimidines **44-46**. The structures of **42-46** were confirmed from their ^1^H-NMR, ^13^C-NMR and mass spectral data. The assignment of the NMR chemical shifts of the thienopyrimidines were made on the basis of COSY ^1^H-NMR and DEPT-135 experiments. HETCOR and HMBC spectra were further used in the structural elucidation of **38, 43, 45** and **46**. Thus, the ^1^H- NMR spectrum of **43** displayed four multiplets and two singlets in the aliphatic region attributable to the protons in five methylenes and two methyl groups. The signals of these aliphatic carbons in the ^13^C-NMR spectrum were verified through the corresponding DEPT-135 technique. The remaining signals in the latter spectrum correspond to the aromatic carbons in **43**, which were unambiguously assigned on the basis of the analysis of HETCOR and HMBC spectral data. Figure 3 shows the HMBC spectrum correlations in **43**.

### 2.2. Biological studies

#### 2.2.1. Antibacterial screening 

Representative microbes [*Staphylococcus aureus* (ATCC 25923), *Klepsela monas*(ATCC 700603), *Pseudomonas aeruginosa* (ATCC 27584) and *Escherichia coli* (ATCC 25922)] were used as test organisms. The filter paper disc method [16,17] was used for the antimicrobial screening of selected compounds (**4a**, **8c, 12**, **15, 17**, **41** and **46**). The tested compounds were dissolved in a suitable solvent (the concentration of the compounds was 10%). Standard blank paper discs (5 mm in diameter) were separately soaked in the solutions of each compound and after 3-5 min transferred onto the surface of growth media seeded with the test organism. After an incubation period under suitable conditions for the test organism (35 °C) and after 24 h, the diameter of the inhibition zones around the discs were measured in millimeters. The effects were compared with the reference antibiotics vancomycin (VA) in the case of *S. aureus* and cefatzidine (CAZ) in the case of other tested organisms. The antibiotics VA and CAZ were used at a concentration of 20 mg/mL as references. The obtained results are summarized in Table 2. Most of the compounds (except **17**) showed very good activity against *Staphylococcus aureus* comparing with the used reference standards. Some of the tested compounds showed very weak or moderate activities aginst *Klepsela monas*, *Pseudomonas aeruginosa* and *Escherichia coli*, as can be noticed from Table 2.

#### 2.2.2. Antitumor studies

The synthesized thienopyrimidines **4a**, **7d**, **8a**, **12**, **15**, **29** and **42** were tested against the following human tumor cell lines: colorectal carcinoma (HCT116), hepatocellular carcinoma (HEPG2), cervix adenocarcinoma (HELA), larynx carcinoma (HEP2), human breast adenocarcinoma (MCF7). The drug doxorubicin [18] has been used as a reference in the present study to compare the inhibition effect for tested compounds on the growing cancer cells. Measurement of Potential Cytotoxicity by the SRB Assay was used [19]. Compounds **8**, **12** and **29** showed very good anticancer activity against HEPG2, while most of the compounds were very active against HELA, except **4a** and 7d. Compounds **7d** and **15** were active toward MCF7. For cell line HCT116 only **7d** and **42** were very active, on the other hand **7d, 8a** and **15** were very active against HEP2. Results are summarized in Table 3.

## 3. Experimental

### 3.1. General

Melting points were determined on an Electrothermal IA9000 series digital capillary melting point apparatus. IR spectra were run (KBr discs) on a Shimadzu FT spectrophotometer 1000. ^1^H- and ^13^C- NMR spectra were recorded in DMSO-d_6_ (or in CDCl_3_) on a JEOL ECP 400 NMR spectrometer operating at 400/100 MHz, with TMS as internal standard. DEPT and HETCOR experiments were recorded on a 500 MHz instrument (Bruker, J.F.B. 288) at King Saud University (Pharmacy Research Centre). Chemical shifts are given in δ (ppm) and coupling constants (*J*) are given in Hz. The assignments of all carbons are made by comparison to ^13^C-NMR spectra of structurally related compounds [12,13] and theoretical grounds [20,21], and with the aid various modern NMR techniques in many cases. Electron impact (EI) MS spectra were recorded on a Shimadzu GCMSQP5050A spectrometer (DB-1 glass column 30 m × 0.25 mm, ionization energy 70 eV), at the Chemistry Department, College of Science, King Saud University. Antimicrobial and anti-cancer tests of some of the synthesized compounds were run in King Abdul Aziz Hospital for the National Guard, Al-hasa, Saudi Arabia and the Pharmacology Unit, National Cancer Institute, Cairo University, Egypt, respectively. The reactions were monitored by TLC, and the purity of the compounds were routinely checked by TLC silica gel plates while the spots were visualised by UV (Uvitec). The starting materials, ethyl 2-amino-4,5-disubstituted thiophene-3-carboxylates **1a-d** and the 2-amino-4,5-disubstituted thiophene-3-carbonitrile **32**, were prepared by condensation of ketones, elemental sulfur, ethyl cyanoacetate or malononitrile as described [14,15].

### 3.2. General procedure for synthesis of **2a,b**

Compounds **2a,b** were both prepared according to a method reported in the literature [22] for similar compounds. 5-Chlorovaleryl chloride (1.3 mL, 10 mmol) was added to a solution of amino ester **1a,b** (10 mmol) in chloroform (20 mL) and the solution was refluxed for 4 h. After cooling, the solution was concentrated under reduced pressure to give a dark oil. Addition of a small amount of water and ethanol yielded a solid that was collected, dried and recrystallized from ethanol.

*2-(5-Chloropentanoylamino)-4,5,6,7-tetrahydrobenzo[b]-thiophene-3-carboxylic acid ethyl ester* **(2a**). Colorless crystals, m.p. 54–55 °C; yield 86%; IR (cm^-1^): 3,230 (NH), 1,678 (CO, ester), 1,662 (CO, amide); ^1^H-NMR (CDCl_3_) δ: 1.36 (3H, t, *J* = 7.3 Hz, CH_2_CH_3_), 1.76 (4H, m, 2CH_2_), 1.86 (4H, m, 2CH_2_), 2.48 (2H, t, *J* = 7.0 Hz, CH_2_-CO), 2.61 (2H, t, *J* = 5.5 Hz, CH_2_), 2.74 (2H, t, *J* = 5.5 Hz, CH_2_), 3.54 (2H, t, *J* = 7 Hz, CH_2_Cl), 4.29 (2H, q, *J* = 7.3 Hz, CH_2_CH_3_), 11.01 (s,NH); ^13^C-NMR (CDCl_3_) δ: 22.61, 26.92, 26.44, 31.89 (4C, 4CH_2_), 23.05, 24.71, 35.90, 44.47 (4C, CH_2_) 14.38 (CH_2_CH_3_), 60.54 (CH_2_CH_3_), 111.46, 126.75, 130.78, 147.54 (4C, thiophene carbons), 166.75 (CO-NH), 169.32 (ester CO).

*2-(5-Chloropentanamido)-5,6,7,8-tetrahydro-4H-cyclohepta[b]thiophene-3-carboxylic acid ethyl ester* (**2b**). Colorless crystals, m.p. 50 °C; yield 86%; IR (cm^–1^): 3,363 (NH), 1,697 (CO, ester), 1,681 (CO, amide); ^1^H-NMR (CDCl_3_) δ: 1.35 (3H, t, *J* = 7 Hz, CH_2_CH_3_), 1.60 (4H, m, 2CH_2_), 1.83 (4H, m, 2CH_2_), 2.45 (2H, t, *J* = 6.9 Hz, CH_2_-CO), 2.67 (2H, m, CH_2_), 2.99 (2H, m, CH_2_), 3.52 (2H, t, *J* = 6.9 Hz, CH_2_Cl), 4.29 (2H, q, *J* = 7 Hz, CH_2_CH_3_), 11.18 (1H, s, NH); ^13^C-NMR (CDCl_3_) δ: 22.63, 26.99, 27.86, 31.87, 32.24 (5C, 5CH_2_), 28.31, 28.62, 35.89, 44.46 (4C, CH_2_) 14.38 (CH_2_CH_3_), 60.72 (CH_2_CH_3_), 112.82, 130.95, 136.36, 145.45 (4C, thiophene carbons), 169.29 (CO), 166.78 (CO-NH).

### 3.3. 2-[5-(4-(2-Methoxyphenyl)-1-piperazin-1-yl]pentanoylamino)-4,5,6,7-tetrahydrobenzo[b]-thio-phene-3-carboxylic acid ethyl ester (**3a**)

A mixture of **2a** (3.9 mmol), 1-(2-methoxyphenyl)piperazine (750 mg, 3.9 mmol), and potassium carbonate (540 mg, 3.9 mmol) was dissolved in DMF (7 mL) and refluxed under stirring for 2 h. After cooling, the suspension was filtered and the solution was extracted with chloroform and washed with water. The organic layers were dried over anhydrous sodium sulfate and evaporated under reduced pressure. Recrystallization from ethanol gave white crystals, m.p. 105–106 °C; yield 94%; IR (cm^–1^): 3,261 (NH), 1,691 (CO, ester), 1,660 (CO, amide); ^1^H-NMR (CDCl_3_) δ: 1.34 (3H, t, *J* = 7.32 Hz, CH_2_CH_3_), 1.60 (2H, m, CH_2_), 1.75 (6H, m, 3 CH_2_), 2.43 (4H, t, *J* = 7.3 Hz, 2 CH_2_-N piperazine), 2.48 (4H, t, *J* = 7.3 Hz, 2 CH_2_-N piperazine), 2.61 (6H, br, 3 CH_2_), 2.73 (2H, br s, CH_2_-CO), 3.81 (3 H, s, O-CH_3_), 4.28 (2H, q, *J* = 7.3 Hz, CH_2_CH_3_), 6.80–6.97 (4H, m, Ar-H), 11.01 (s, NH); ^13^C-NMR (CDCl_3_) δ: 22.95, 23.07, 23.40, 24.42 (4C, 4 CH_2_ N-piperazine), 26.46, 36.80, 50.72, 53.55 (4C, 4 CH_2_), 55.40 (OCH_3_), 26.46 (2 C), 58.26 (2 C) (4C, 4 CH_2_), 14.41 (CH_2_CH_3_), 60.50 (CH_2_CH_3_), 111.30, 126.60, 130.71, 147.74 (4 C, thiophene carbons), 111.19, 118.22, 121.02, 122.88, 141.46, 152.32 (phenyl carbons), 166.75 (CO-NH), 169.32 (CO).

### 3.4. 2-(5-(4-(2-Methoxyphenyl)piperazin-1-yl)pentanamido)-5,6,7,8-tetrahydro-4H-cyclohepta[b]-thiophene-3-carboxylic acid ethyl ester (**3b**)

Compound **3b** was prepared from equimolar amounts of **2b** and 1-(2-methoxyphenyl)piperazine following the same conditions and work up described for the preparation of **3a**. The product could not be induced to crystallize, although the NMR spectra proved the structure. Yield 85%; ^1^H-NMR (CDCl_3_) δ: 1.72 (4H, m, 2CH_2_), 1.84 (2H, m, CH_2_), 2.55 (2H, m, CH_2_), 2.85 (2H, m, CH_2_); ^13^C-NMR (CDCl_3_) δ: 19.64, 26.20, 28.62, 29.15 (2C) (cycloheptane carbons).

### 3.5. 3-Amino-2-[4-[4-(2-methoxyphenyl)-piperazin-1-yl]butyl]-5,6,7,8-tetrahydro-3H-benzo[4,5]-thieno[2,3-d]pyrimidin-4-one (**4a**) 

A mixture of **3a** (20 mmol) and hydrazine hydrate (4 mL, 80 mmol) was refluxed for 12 h in ethanol (10 mL). After cooling, the product was collected and washed with ethanol, dried and recrystallized from ethanol, to yield **4a** colorless crystals, m.p. 163–164 °C; yield 92%; IR (cm^–1^): 3,273 and 3,145 (NH), 1,672 (C=O); ^1^H and ^13^C-NMR data: see Table 1; MS: *m/z* 467 (M^+^, 2.02%), 452 (M^+^-CH_3_), 18.27%), 204 (M^+^-(NH_2_, (CH_2_)_4_-N_2_C_4_H_8_-C_6_H_4_-OCH_3_)), 24.29%), 119 (M^+^= [C_6_H_4_-N-CH_2_CH_3_]^+^, 100%).

### 3.6. 3-Amino-2-[4-[4-(2-methoxyphenyl)-piperazin-1-yl]butyl]-6,7,8,9-tetrahydro-3H,5H-[4,5]thieno[2,3-d]pyrimidin-4-one **(4b**)

This compound was prepared from **3b** following the same conditions and work up described for the preparation of **4a**. Colorless crystals, m.p. 183–185 °C; yield 81%; IR (cm^–1^): 3310, 3135 (NH), 1670 (C=O); ^1^H and ^13^C-NMR data: almost the same data as **4a** above except for an additional CH_2_ in the cycloheptane moiety in **4b**: δ_H_ 1.79 (4H, m, 2 CH_2_), 1.95 (2H, m, CH_2_), 2.51 (2H, m, CH_2_), 2.87 (2H, m, CH_2_) and δ_C_ 19.81, 25.14, 27.40, 29.15 (2C, cycloheptane carbons); MS: *m/z* 481 (M^+^, 7.3%), 466 (M^+^ - CH_3_), 15.39%).

### 3.7. 2-(3′-Ethoxycarbonyluriedo)-3-ethoxycarbonyl-5,6,7,8-tetrahydro-4H-cyclohepta[b] thiophene (**5**) 

A mixture of **1b** (1.195 g, 5 mmol) and ethyl isocyanatoformate (575 mg, 5 mmol) was placed in a 50 mL conical flask covered with a funnel glass and then irradiated with microwaves (950 W) for 35 seconds. The cold reaction mixture was treated with ethanol and the solid product was filtered and recrystallized from ethanol-water to give colorless needles, m.p. 167–169 °C; yield 88%; IR (cm^–1^): 3,207, 3,115 (2 NH), 1,739 (CO, ester), 1,678, 1,675 (2 CO, amide); ^1^H-NMR (CDCl_3_) δ: 1.32 (3H, t, CH_3_), 1.36 (3H, t, CH_3_), 1.64 (4H, m, 2 CH_2_), 1.83 (2H, m, CH_2_), 2.71 (2H, t, CH_2_), 3.05 (2H, t, CH_2_), 4.30 and 4.40 (each 2H, q, O-CH_2_), 7.45 (1H, s, NH), 12.43 (1H, s, NH); ^13^C-NMR (CDCl_3_) δ: 14.34 and 14.44 (each 3H, t, CH_3_), 27.06, 27.89, 28.35, 28.69, 32.33 (5C-5CH_2_), 60.72, 62.98 (2C, O-CH_2_), 114.22, 131.20, 137.18, 144.16 (thiophene ring), 165.52 (CO), 153.11, 149.56 (2 CO-amide); MS: *m/z* 354 (M^+^, 89.5%), 265 (M^+^-NHCO_2_C_2_H_5_,H, 23%), 192 (M^+^-NHCO_2_C_2_H_5_,H, CO_2_C_2_H_5_, 100%), 164 (M^+^-NHCO_2_ C_2_H_5_, H, CO_2_C_2_H_5_, CO, 74%).

### 3.8. 1,5,6,7,8-Hexahydro-3H-cyclohepta[4,5]thienopyrimidin-2,4-dione (**6**)

Compound **5** (1770 mg, 5 mmol) was added to a solution of sodium ethoxide [sodium metal (120 mg), absolute ethanol (15 mL)], and refluxed 0.5 h, then the solvent was removed under reduced pressure. The residue was treated with water, then acidified with dil. HCl 1:1 (pH = 4), and the solid formed was collected and recrystallized from ethanol to give **6** as colorless crystals, m.p. > 300 °C; yield 82%; IR (cm^–1^): 3,095, 3,153 (2 NH), 1,708, 1,666 (2 CO), 2,600–3,300 (OH); ^1^H-NMR (DMSO-d_6_) δ: 1.53 (4H, m, 2CH_2_), 1.80 (2H, m, CH_2_), 2.69 (2H, t, CH_2_), 3.10 (2H, t, CH_2_), 10.95 (s, NH), 11.75 (NH); ^13^C-NMR (DMSO-d_6_) δ: 27.21, 27.31, 27.98, 28.87, 32.41 (5C, 5 CH_2_), 113.92, 129.56, 136.79, 149.83 (thiophene carbons), 150.87, 160.73 (2 CO); MS: *m/z* 236 (M^+^, 100%), 208 (M^+^-C_2_H_4_, 39.4%), 193 (M^+^-C_2_H_4_,NH), 36%), 165 (M^+^-C_2_H_4_,NH, CO), 48%), 137 (M^+^-C_2_H_4_, NH, 2CO), 42.4%), 133 (M^+^-(C_2_H_4_, NH, 2CO, H), 33.3%).

### 3.9. General procedure for synthesis of 5,6-disubstituted-3H-thieno[2,3-d] pyrimidin-4-ones **7a-d**

A mixture of **1a-d** (2 mmol) and formamide (20 mL) was heated under reflux for 1.5 h, then left to cool to room temperature overnight. The solid formed was filtered, washed with water, dried and recrystallized from ethanol [13].

*5,6,7,8-Tetrahydro-3H-benzo[4,5]thieno[2,3-d]pyrimidin-4-one* (**7a**). Fine brown needles, m.p. 224–226 °C; yield 92%; IR (cm^–1^): 3,157 (NH), 3,007 (C-H), 1,658 (CO), 1,589 (C=C); ^1^H-NMR (DMSO-d_6_) δ: 1.76 (4H, m, 2 CH_2_), 2.71 (2H, t, CH_2_), 2.86 (2H, t, CH_2_), 7.98 (H-2), 12.29 (br, s, NH); ^13^C-NMR (DMSO-d_6_) δ: 22.31, 23.00, 24.98, 25.88 (aliphatic carbons), 123.23, 131.35, 132.64, 145.37 (4C, thiophene carbons), 158.21 (C=N), 162.96 (C=O); MS: *m/z* 206 (M^+^, 20%), 178 (M^+^-(HCNH), 1%), 57 (C_2_SH, 100%). 

*3,5,6,7,8,9-Hexahydrocyclohepta[4,5]thieno[2,3-d]pyrimidin-4-one* (**7b**). Fine brown crystals, m.p. 118–220 °C; yield 90%; IR (cm^–1^): 3,151 (NH), 3,012 (C-H), 1,656 (CO), 1,598 (C=C); ^1^H-NMR (DMSO-d_6_) δ: 1.60 (4H, m, 2 CH_2_), 1.84 (2H, m, CH_2_), 2.82 (2H, m, CH_2_), 3.25 (2H, t, CH_2_), 7.98 (H-2), 12.30 (br, s, NH); ^13^C-NMR (DMSO-d_6_) δ: 27.70 (2C), 27.81, 29.57, 32.50 (aliphatic carbons), 123.78, 136.98, 145.07, 149.00 (4C, thiophene carbons), 158.75 (C=N), 161.40 (C=O); MS: *m/z* 220 (M^+^, 63%), 192 (M^+^-C_2_H_4_, 45%), 165 (M^+^-C_2_H_4_,HCN, 39%), 122 (M^+^-C_2_H_4_,HCN, CO, NH, 27%), 58, C_2_H_2_S, 100%). 

*5,6-Dimethylthieno[2,3-d]pyrimidin-4-one* (**7c**). Fine yellow crystals, m.p. 269–270 °C; yield 93%; IR (cm^–1^): 3,151 (NH), 3,057 (C-H), 1,656 (CO), 1,558 (C=C); ^1^H-NMR (DMSO-d_6_) δ: 2.33 (3H, s, CH_3_), 2.37 (3H, s, CH_3_), 7.98 (1H, s, CH), 12.29 (br, s, NH); ^13^C-NMR (DMSO-d_6_) δ: 13.09, 13.40 (2C,CH_3_), 129.25, 129.79, 132.64, 145.23 (4C, thiophene carbons), 158.49 (C=N), 162.19 (C=O); MS: *m/z* 180 (M^+^, 100%), 165 (M^+^-CH_3_, 1%), 57 (C_2_SH, 94%). 

*7-Benzyl-5,6,7,8-tetrahydro-3H-pyrido[4′,3′:4,5]thieno[2,3-d]pyrimidin-4-one* (**7d**). Fine yellow crystals, m.p. 234–235 °C; yield 89%; IR (cm^–1^): 3,157 (NH), 3,068 (C-H), 1,660 (CO), 1,581 (C=C); ^1^H-NMR (DMSO-d_6_) δ: 2.75 (2H, t, CH_2_), 2.93 (2H, t, CH_2_), 2.60 (2H, s, CH_2_), 2.69 (2H, s, CH_2_), 7.26–7.35 (5H, m, Ar-H), 8.02 (1H, s, CH), 12.39 (1H, br, s, NH); ^13^C-NMR: (DMSO-d_6_) δ: 26.20, 49.58, 51.51, 61.38 (4C, aliphatic carbons), 127.65, 128.85 (2C), 129.34 (2C), 129.80 (Ar-CH), 122.87, 130.38, 138.69, 145.74 (4C, thiophene carbons), 158.21 (C=N); 163.43 (C=O); Ms: *m/z* 297 (M^+^, 93%), 206 (M^+^-CH_2_-C_6_H_5_, 32%), 178 (M^+^-CH_2_-C_6_H_5_, HCN, H, 100%). 

### 3.10. General procedure for synthesis of 4,5-disubstituted-3H-thieno[2,3-d] pyrimidin-4-thiones **8a,c,d**

A mixture of compound **7a,c,d** (10 mmol), phosphorous pentasulphide (4.02 g, 30 mmol) and dry pyridine (50 mL) was refluxed with stirring for 2 h. The mixture was evaporated to dryness under reduced pressure and the residue was boiled with water (100 mL) for one hour. After cooling overnight in refrigerator, the formed solid was recrystallized from a suitable solvent.

*5,6,7,8-Tetrahydro-3H-benzo[4,5]thieno[2,3-d]pyrimidin-4-thione* (**8a**). Recrystallized from ethanol give yellow crystals, m.p. 235–237 °C; yield 90%; IR (cm^–1^): 3,132 (NH), 3,061 (Ar-CH), 1,562 (C=C), 1,185 (C=S); ^1^H-NMR (DMSO-d_6_) δ: 1.61 (4H, m, 2CH_2_), 2.53 (2H, t, CH_2_), 3.04 (2H, t, CH_2_), 7.62 (1H, s, CH), 13.14 (br, s, NH); ^13^C-NMR (DMSO-d_6_) δ: 22.26, 22.40, 25,63, 27.99 (aliphatic carbons), 132.55, 132.91, 135.68, 142.42 (thiophene carbons); 161.18 (C=N); 178.24 (C=S); MS: *m/z* 222 (M^+^, 100%), 180 (M^+^- NCHNH, 66%), 149 (M^+^-NCHNH, S, +H, 66%). 

*5,6-Dimethyl-3H-thieno[2,3-d]pyrimidin-4-thione* (**8c**). Light brown crystals, m.p. 247–248 °C (ethanol); yield 88%; IR (cm^–1^): 3,120 (NH), 3,068 (Ar-CH), 1,568 (C=C) 1,181 (C=S); ^1^H-NMR (CDCl_3_) δ: 2.32 (3H,s,CH_3_), 2.57 (3H, s, CH_3_), 8.09 (1H, s, CH), 13.56 (br, s, NH); ^13^C-NMR (CDCl_3_) δ: 13.05, 15.05 (2C, CH_3_), 130.29, 131.97 (2C), 143.13 (4C, thiophene carbons), 160.13 (C=N), 178.09 (C=S); MS: *m/z* 196 (M^+^, 100%), 181 (M^+^-CH_3_, 24%), 163 (M^+^-SH, 54%).

*7-Benzyl-5,6,7,8-tetrahydro-3H-pyredo-[4′,3′:4,5]thieno[2,3-d]pyrimidin-4-thione* (**8d**). Yellow powder, m.p. 204–207 °C (ethanol-DMF); yield: 50%; IR (cm^–1^): 3,415 (NH), 3,028 (Ar-CH), 1,180 (C=S), 1,629 (C=C); ^1^H-NMR (DMSO-d_6_) δ: 2.98 (2H, CH_2_), 3.37 (2H, CH_2_), 4.13 (2H, CH_2_), 4.31 (2H, s, CH_2_-Ph), 7.23–7.39 (5H, m, Ar-H), 8,32 (H-2), 13.78 (br.s, NH).

### 3.11. General procedure for the synthesis of **10a,c,d**

A mixture of **1a,c,d** (10 mmol) and ethoxycarbonyl isothiocyanate (10 mmol) in ethanol (5 mL) was placed in a 50 mL conical flask covered with a funnel glass and then irradiated with microwaves (950 W) for 35–40 seconds. The cold reaction mixture was treated with ethanol and the solid product was filtered and recrystallized from a suitable solvent.

*2-(Ethoxycarbonylamino-carbothioyl)amino-4,5,6,7-tetrahydrobenzo[4,5]thiophene- 3-carboxylic acid ethyl ester* (**10a**). Yellow crystals, m.p. 193–195 °C (ethanol); yield 92%; IR (cm^–1^): 3,182 (NH), 1,732 (ester CO), 1,680 (amide CO), 1,562, 1,533 (C=C), 1,147 (C=S), 1,201 (C-O); ^1^H-NMR (CDCl_3_) δ: 1.30 and 1.34 (each 3H, t, *J* = 7.3 Hz, CH_3_CH_2_), 4.32 and 4.40 (each 2H, q, *J* = 7.3 Hz, CH_2_CH_3_), 1.76 (4H, m, 2CH_2_), 2.63 (2H, t, CH_2_), 2.77 (2H, t, CH_2_), 8.19 (br. s, NH), 14.03 (s, NH); ^13^C-NMR (CDCl_3_) δ: 14.36, 14.44 (2C, 2 CH_3_), 22,92 (2C, 2 CH_2_), 24.45, 26.49, 60.81, 63.09, 116.09, 128.31, 131.78, 147.16 (thiophene carbons), 151.41 (CO-N), 165.66 (CO), 173.27 (C=S).

*2-(Ethoxycarbonylamino-carbothioyl)amino-4,5-dimethyl[4,5]thiophene-3-carboxylic acid ethyl ester* (**10c**). Yellow crystals, m.p. 274–176 °C (ethanol); yield 90%; IR (cm^–1^): 3,176 (NH), 1,728 (C=O), 1,683 (amide C=O), 1,564, 1,531 (C=C), 1,170 (C=S), 1,293 (C-O); ^1^H-NMR (CDCl_3_) δ: 1.32 and 137 (each 3H, t, CH_3_CH_­2_), 2.64 (3H, s, CH_3_-C4), 2.69 (3H, s, CH_3_-C5), 4.43 (2H, q, CH_2_-CH_3_), 4.32 (2H, q, CH_2_-CH_3_), 8.07 (IH, s, NH), 14.03 (H, s, NH); ^13^C-NMR (CDCl_3_) δ: 12.46, 14.36, 14.41, 14.57 (4C, 4CH_3_), 60.93, 63.12 (2C, 2CH_2_), 117.24, 125.01, 130.30, 146.23 (4C, thiophene carbons) 151.40 (CO), 165.68 (CO), 173.16 (C=S).

*2-(Ethoxycarbonylamino-carbothioyl)amino-7-benzyl-5,6,7,8-tetrahydro-3H-pyredo-[4′,3′:4,5]thio-phene-3-carboxylic acid ethyl ester* (**10d**). Light yellow powder, m.p. 205–207 °C (ethanol and DMF); yield 91%; IR (cm^–1^): 3,064 (NH), 3,024, 1,728 (ester CO), 1,683 (amide CO), 1,564, 1,525 (C=C), 1,110 (C=S), 1,213 (C-O); ^1^H-NMR (DMSO-d_6_) δ: 1.33 (3H, t, *J* = 7.4 Hz,CH_3_), 1.36 (3H, t, *J* = 7.4 Hz, CH_3_), 4.35 (2H, q, *J* = 7.4 Hz, CH_2_), 4.43 (2H, q, *J* = 7.4 Hz, CH_2_), 2.79 (2H, m, CH_2_), 2.94 (2H, m, CH_2_), 3.57 (2H, s, CH_2_), 3.71 (2H, s, CH_2_), 8.14 (NH-CO), 14.09 (NH); ^13^C-NMR (DMSO-d_6_) δ: 14.37 (CH_3_), 14.44 (CH_3_), 60.92 and 63.19 (2CH_2_-O), 27.01, 50.32, 51.36, 62.16 (CH_2_-N), 115.61, 125.48, 130.35, 147.91 (4C, thiophene carbons), 127.37, 128.46 (2C), 129.24 (2C), 138.12, 151.42 (amide CO), 165.52 (ester CO), 173.36 (C=S).

### 3.12. General procedure for the synthesis of **11a,c,d**

These susbstances were prepared according to a method reported in the literature [23]. Compound **10** (1 mmol) was dissolved in solution of sodium ethoxide (230 mg sodium and 15 mL of absolute ethanol) and the solution was heated under reflux for 30 min. The solvent was then evaporated under vacuum, some water was added to the residue, and the pH of the mixture was adjusted to 4 with hydrochloric acid. The product that separated was collected and crystallized from a suitable solvent.

*2-Thioxo-2,3,5,6,7,8-hexahydro-1H-benzo[4,5]thieno[2,3-d]pyrimidin-4-one* (**11a**). White powder, m.p. 275–277 °C (ethanol); yield 88%; IR (cm^–1^): 3,157 (NH), 1,672 (CO), 1,577, 1,556 (C=C), 1,157 (C=S); ^1^H-NMR (DMSO-d_6_) δ: 1.78 (4H, m, 2CH_2_), 2.70 (2H, m, CH_2_), 2.80 (2H, m, CH_2_), 12.34 (s, NH), 13.36 (s, NH); ^13^C-NMR (DMSO-d_6_) δ: 22.13, 23.08, 24.50, 25.47 (4C, 4 CH_2_), 117.04, 128.78, 131.43, 150.54 (4C, thiophene carbons), 157.58 (CO), 173.40 (C=S);

*5,6-Dimethyl-2-thioxo-2,3-dihydro-1H-thieno[2,3-d]pyrimidin-4-one* (**11c**). Light yellow powder, m.p. 205–207 °C (ethanol); yield 82%; IR (cm^–1^): 3,427, 3,404 (NH), 1,664 (CO), 1,604, 1,556 (C=C), 1,174 (C=S); ^1^H-NMR (DMSO-d_6_) δ: 2.20 (3H, s, CH_3_), 2.24 (3H, s, CH_3_), 12.26 (s, NH), 13.26 (s, NH); ^13^C-NMR (DMSO-d_6_) δ: 12.49, 13.01 (2C, 2CH_3_), 117.71, 125.68, 129.21, 149.79 (4C, thiophene carbons), 157.71 (CO), 173.16 (C=S).

*7-Benzyl-2-thioxo-2,3,5,6,7,8-hexahydro-1H-pyrido[4′,3′:4,5]thieno[2,3-d]pyrimidin-4-one* (**11d**). Orange powder, m.p. >300 °C (ethanol and DMF); yield 20%**;** IR (cm^–1^): 3,350, 3,180 (NH), 3,076, 1,681 (CO), 1,544, 1,519 (C=C), 1,130 (C=S); ^1^H-NMR (DMSO-d_6_) δ: 3.15 (2H, CH_2_), 3.39 (2H, CH_2_), 4.24 (2H, CH_2_), 4.43 (2H, s, CH_2_-Ph), 7.26-7.38 (5H, m, Ar-H), 11.40 (br. s, NH), 12.50 (s, NH).

### 3.13. Synthesis of **12** and **13**

To a solution of **11a** (1.19 g, 5 mmol) was added aroyl hydrazine (5 mmol) in *n*-butanol (15 mL) and the mixture was heated under reflux for 20 h. The solid obtained was cooled, collected and recrystallized from butanol.

*1-(4-Chlorophenyl)-6,7,8,9-tetrahydro-4H-benzo[4,5]thieno[2,3-d][1,2,4]triazolo[3,4-b]-pyrimidin-5-one* (**12**): Fine yellow crystals, m.p. 218–219 °C; yield 86%; IR (cm^–1^): 3,253 (NH), 3,035 (Ar-CH), 1,656 (CO), 1,600 (C=N), 1,566 (C=C); ^1^H-NMR (DMSO-d_6_) δ: 1.70 (4H, 2 CH_2_), 2.63 (2H, m, CH_2_), 2.73 (2H, m, CH_2_), 7.52 (2H, d, *J* = 8.8 Hz, C_6_H_4_), 7.82 (2H, d, *J* = 8.8 Hz, C_6_H_4_), 9.87 (br. s, NH); ^13^C-NMR (DMSO-d_6_) δ: 22.05, 22.00, 24.42, 25.39 (aliphatic carbons), 116.97, 128.70, 131.36, 150.56 (4C, thiophene carbons), 128.96 (2C), 129.41 (2C), 136.43 (C-Cl), 132.56 (C, Ar-C^1^), 157.82 (C=N), 165.34 (C=N), 173.31 (C=O); MS: *m/z* 356 (M^+^, 1.1%), 220 (M^+^-Cl-C_6_H_4_-CN) + H, 9%), 193 ( M^+^-Cl-C_6_H_4_-CN, C_2_H_4_ + 2H, 13%).

*1-(3-Nitrophenyl)-6,7,8,9-tetrahydro-4H-benzo[4,5]thieno[2,3-d][1,2,4]triazolo[3,4-b]-pyrimidin-5-one* (**13**): Fine yellow powder, m.p. 234–235 °C; Yield 79%; IR (cm^–1^): 3,251 (NH), 3,107–3,041, 1,687 (CO), 1,650 (C=N), 1,579 (C=C), 1,523, 1,346 (N=O); ^1^H-NMR (DMSO-d_6_) δ: 1.70 (4H, 2CH_2_), 2.60 (2H, m, CH_2_), 2.71 (2H, m, CH_2_), 7.73 (1H, t, *J* = 8 Hz, Ar-^5^CH-*m*), 8.22 (1H, d, *J* = 6.0 Hz, Ar-^6^CH-*o*), 8.33 (1H, d, *J* = 6.0 Hz, Ar-^4^CH-*p*), 8.60 (1H, s, Ar-^2^CH-*o*), 12.20 (s, NH); ^13^C-NMR (DMSO-d_6_) δ: 21.24, 22.37, 23.78, 24,73 (4C, aliphatic carbons), 116.36, 128.01, 130.77, 150.00 (4C, thiophene carbons), 121.66, 125.45, 129.97, 133.10 (4C, Ar-CH), 134.71 (C), 147.78 (C-NO_2_), 156.81 (C=N), 163.47 (C=N), 172.86 (C=O); MS: *m/z* 367 (M^+^, 87%), 221 (M^+^- (N=C-C_6_H_3_-NO_2_ )+2H, 24%), 207 ((M^+^- [(N=C-C_6_H_3_-NO_2_ ) + 2H]-NH_2_, 20%).

### 3.14. 2-Hydrazino-5,6,7,8-tetrahydro-3H-benzo[4,5]thieno[2,3-d]pyrimidin-4-one (**14**) 

A mixture of **11a** (950 mg, 4 mmol) and 99% hydrazine hydrate (4 mL, 80 mmol) in pyridine (20 mL) was heated under reflux for 15 h. The mixture was evaporated under reduced pressure and the residue was treated with ethanol. The solid product was filtered and washed several times with ethanol to give colorless crystals, m.p. 265–267 °C; yield 83%; IR (cm^–1^): 3,319, 3,265 (NH, NH_2_), 1,658 (CO), 1,597 (C=C); ^1^H-NMR (DMSO-d_6_) δ: 1.73 (4H, m, 2CH_2_), 2.50 (2H, m, CH_2_), 2.75 (2H, m, CH_2_), 8.20 (NH), NH_2_ not observed; ^13^C-NMR (DMSO-d_6_) δ:22.49, 23.30, 24.73, 25.84 (aliphatic carbons), 114.25, 125.12, 130.60, 155.67 (thiophene carbons), 158.36 (C=N), 167.14 (CO) [13].

### 3.15. 5,6,7,8-Tetrahydro-1H-benzo[4,5]thieno[2,3-d][1,2,4]triazolo[3,4-a]-pyrimidin-4-one (**15**)

A mixture of **14** (1.18 g, 5 mmol) and excess triethyl orthoformate (10 mL) was heated under reflux with stirring for 2 h. The excess of the orthoformate was removed under reduced pressure. The formed solid was collected, washed with ethanol, dried and recrystallized from ethanol to give a yellow powder, m.p. 277–279 °C; yield 84%; IR (cm^–1^): 3,118 (NH), 3,039, 1,672 (CO), 1,608 (C=N), 1,554 (C=C); ^1^H-NMR (DMSO-d_6_): δ 1.77 (4H, 2CH_2_), 2.62 (2H, CH_2_),2.83 (2H, CH_2_), 9.01 (1H, s, CH), 14.04 (1H, s, NH); ^13^C-NMR (DMSO-d_6_) δ: 22.40, 23.17, 24,91, 25.94 (4C, aliphatic carbons), 112.08, 126.64, 130.19, 148.87 (4C, thiophene carbons), 132.50 (CH, triazole), 151.93 (C=N), 167.82 (C=O); MS: *m/z* : 246 (M^+^, 87%), 245 (M^+^-H, 43%), 195(M^+^-HCN, C_2_H_4_ + 4H, 37%).

### 3.16. 3-Methyl-5,6,7,8-tetrahydro-1H,2H-benzo[4,5]thieno[2,3-d][1,2,4]triazolo[3,4-a]-pyrimidin-4-one (16) 

A mixture of **14** (1.18 g, 5 mmol) and excess triethyl orthoacetate (10 mL) was heated under reflux with stirring for 2 h. The solvent was removed under reduced pressure, The formed solid was collected washed with ethanol, dried and recrystallized from ethanol, give a white powder, m.p. 292–294 °C; yield 79%; IR (cm^–1^); 3,232 (NH), 1,714 (CO), 1,701 (C=N), 1,620 (C=C); ^1^H-NMR (DMSO-d_6_): δ 2.98 (3H, s, CH_3_), 1.68 (4H, 2CH_2_), 2.68 (2H, CH_2_), 2.76 (2H, CH_2_), 12.30 (1H, s, NH); MS *m/z*: 260 (M^+^, 100%), 245 (M^+^-CH_3_, 15%), 232 (M^+^-(C_2_H_4_), 57%). 

### 3.17. 3-Thioxo-5,6,7,8-tetrahydro-1H,2H-benzo[4,5]thieno[2,3-d][1,2,4]triazolo[3,4-a]-pyrimidin-4-one (17)

A mixture of **14** (1.18 g, 5 mmol) in pyridine (15 ml) and carbon disulfide (380 mg, 5 mmol) was heated under reflux for 5h. After cooling, the obtained solid was collected and recrystallized from acetic acid to give a white powder, m.p. >300 °C; yield 81%; IR (cm^–1^): 3,105 (br, NH), 2,945, 1,664 (CO); ^1^H-NMR (DMSO-d_6_) δ: 1.75 (4H, 2CH_2_), 2.73 (2H, br, CH_2_), 2.83 (2H, br, CH_2_), 12.57 (br. s, NH), 13.86 (br. s, NH); ^13^C-NMR (DMSO-d_6_) δ: 22.19, 22.92, 24.41, 25.16 (4C, aliphatic carbons), 118.33, 130.93, 131.72, 139.32 (4C, thiophene carbons), 144.98 (C=N), 157.71 (C=O), 158.81 (C=S); MS: *m/z* 278 (M^+^, 100%), 250 ( M^+^-C_2_H_4_, 43%), 245 (M^+^-SH , 31%).

### 3.18. Synthesis of disubstituted thienyl-2-thioureides **20-23**

#### 3.18.1. Method A

2-Amino-3-ethoxycarbonyl thiophene **1a,d** (100 mmol) was dissolved in hot ethanol (100 mL) and phenyl (or *p*-clorophenyl) isothiocyanate (110 mmol) was added dropwise with stirring. The reaction mixture was heated under reflux on water bath for 2 h, then left to cool overnight and the separated crude solid was filtered, washed with ethanol, and recrystallized from ethanol [12]. 

#### 3.18.2. Method-B

A mixture of **1a,d** (20 mmol) and phenyl (or *p*-clorophenyl) isothiocyanate (20 mmol) placed in a 50 mL conical flask covered with a funnel glass and then irradiated with microwaves (950 W) for 35–40 seconds. The cold reaction mixture was then treated with ethanol and the solid product was filtered off and recrystallized from ethanol [13].

*2-(3-Phenylthioureido)-4,5,6,7-tetrahydrobenzo[b]thiophene-3-carboxylic acid ethyl ester* (**20**). Fine colorless needles, m.p. 187–189 °C; yield: (78%)^A^, (89%)^B^; IR (cm^–1^): 3,196 (NH), 3,032 (Ar-CH), 1,656 (CO), 1,554 (C=C), 1,195 (C=S); ^1^H-NMR (CDCl_3_) δ: 0.97 (3H, *J* = 7.3 Hz, t, CH_2_CH_3_), 1.43 (4H, m, 2CH_2_), 2.27 (2H, m, CH_2_), 2.41 (2H, m, CH_2_), 3.87 (2H, *J* = 7.3 Hz, q, CH_2_-CH_3_), 6.89 (t, *J* = 7.32 Hz, Ar-CH(*p*)), 7.05 (2H, t, *J* = 7.32 Hz, Ar-CH(*m*)), 7.11 (2H, d, *J* = 8.08 Hz, Ar-CH(*o*)), 10.03 (s, NH), 11.70 (s, NH); ^13^C-NMR (CDCl_3_) δ: 14.25 (CH_3_), 22.92, 23.02, 24,09, 26.17 (aliphatic carbons); 60.14 (O-CH_2_), 112.01, 125.89, 130.35, 150.17 (4C, thiophene carbons); 124.51, 125.89(2C), 128.94 (2C), 137.81 (aromatic carbons), 166.13 (C=O); 175.99 (C=S); MS: *m/z* : 360 (M^+^, 20%), 326 (M^+^-SH, H, 17%), 225 (M^+^- (SH, H, C_6_H_5_, CN) + 2H, 33%). 

*2-(3-(4-Chlorophenyl)thioureido)-4,5,6,7-tetrahydrobenzo[b]thiophene-3-carboxylic acid ethyl ester* (**21**). Yellow needles, m.p. 198–199 °C; yield (71%)^A^, (88%)^B^; IR (cm^–1^): 3,141 (NH), 3,078 (Ar-CH), 1,652 (CO), 1,558 (C=C), 1,195 (C=S); ^1^H-NMR (CDCl_3_) δ: 1.25 (3H, t, CH_2_CH_3_), 1.75 (4H, m, 2CH_2_), 2.60 (2H, m, CH_2_), 2.70 (2H, m, CH_2_), 4.15 (2H, q, CH_2_CH_3_), 7.28 (2H, d, *J* = 8.8 Hz, Ar-CH), 7.41 (2, d, *J* = 8.8 Hz, Ar-CH), 7.92 (br. s, NH), 12.25 (1H, s, NH); ^13^C-NMR (CDCl_3_) δ: 14.12 (CH_3_), 22.92, 23.02, 24.40, 26.38 (4C, aliphatic carbons), 60.65 (O-CH_2_), 113.23, 127.28, 133.18, 149.81 (thiophene carbons), 126.80 (2C), 130.13 (2C), 130.96, 134.66 (phenyl carbons), 166.68 (C=O), 176.06 (C=S); MS: *m/z* 394 (M^+^, 26%) 396 (M^+^+2, 15%), 225 (M^+^-(OCH_2_CH_3_,NH-C_6_H_4_Cl) + 2H, 39%), 179 (M^+^-(OCH_2_CH_3_,NHCSNH-C_6_H_4_Cl), 100%).

*6-Benzyl-2-(3-phenylthioureido)-4,5,6,7-tetrahydrothieno[2,3-c]pyridine-3-carboxylic acid ethyl ester* (**22**). Orange needles, m.p. 274–276 °C; yield (64%)^A^,(77%)^B^; IR (cm^–1^): 3,196 (br, NH), 3,032 (Ar-CH), 1,656 (CO), 1,554 (C=C), 1,197 (C=S); ^1^H-NMR (CDCl_3_) δ: 1.21 (3H, *J* = 7.3 Hz, t, CH_2_CH_3_), 2.93 (4H, m, 2CH_2_), 3.37 (4H, br. s, 2CH_2_), 4.12 (2H, *J* = 7.3 Hz, q, CH_2_CH_3_), 7.32 (5H, m, C_6_H_5_), 7.46 (5H, m, C_6_H_5_), 7.95 (s, NH), 12.11 (s, NH); MS: *m/z* 450 (M^+^-H, 16%), 91 ((Ph-CH_2_), 100%).

*6-Benzyl-2-(3-(4-chlorophenyl)thioureido)-4,5,6,7-tetrahydro thieno* *[2,3-c]pyridine-3-carboxylic acid ethyl ester* (**23**). Yellow needles, m.p. 259–261 °C; yield (54%)^A^, (69%)^B^; IR (cm^–1^): 3,176 (NH), 3,053, 3,031 (Ar-CH), 1,652 (CO), 1,558 (C=C), 1,197 (C=S); ^1^H-NMR (CDCl_3_) δ: 1.24 (3H, t, *J* = 7.36 Hz, CH_2_CH_3_), 2.85 (4H, m, 2CH_2_), 3.57 (2H, s, CH_2_), 3.74 (2H, s, CH_2_), 4.17 (2H, q, *J* = 7.36 Hz, CH_2_CH_3_), 7.22 (2H, d, *J* = 8.8 Hz, Ar-CH), 7.38 (2H, d, *J* = 8.8 Hz, Ar-CH), 7.24–7.39 (5H, m, C_6_H_5_), 8.37 (br. s, NH), 12.15 (s, NH); ^13^C-NMR (CDCl_3_) δ: 14.25 (CH_3_), 26.46, 50.13, 51.02, 61.85 (4C, CH_2_); 60.77 (O-CH_2_), 112.56, 127.12, 133.19, 150.65 (4C, thiophene ring); 123.90, 126.85 (2C), 130.09 (2C), 134.62 (C_6_H_5_), 126.87 (2C), 128.52 (2C), 129.17, 129.41 (*p*-Cl-C_6_H_4_), 166.35 (C=O), 176.03 (C=S); MS: *m/z* 485 (M^+^, 0.02%), 316 (M^+^-(NH-C_6_H_4_Cl,OCH_2_CH_3_) +H, 9%), 168 (M^+^- (NH-C_6_H_4_Cl,OCH_2_CH_3_, CH_2_C_6_H_5_, NCH_2_,CO), 80%). 

### 3.19. General procedure for the preparation of the monopotassium salts of **24** and **25**

A mixture of the compounds **21**, **22** (13.5 mmol) and potassium hydroxide (760 mg, 13.5 mmol) in absolute ethanol (55 mL) was heated under reflux with stirring for 1h. The suspension was filtered while hot, and the solid was washed with hot absolute ethanol to give **24**) [13] and **25** which were both used without any further purification.

### 3.20. General procedure for the preparation of **18a,d**

#### 3.20.1. Method A

A suspension of potassium salts of **24**, **25** in water (50 mL) was acidified with concentrated hydrochloric acid and stirred at room temperature for 30 min. The solid was collected by filtration, washed with water and recrystallized from the suitable solvent to give **18a,d**.

#### 3.20.2. Method B: Synthesis of **18d**

A mixture of **1d** (3.16 g, 10 mmol) and phenyl isothiocyanate (1350 mg, 10 mmol) in acetonitrile (30 mL) was heated under reflux for 15 h in the presence of anhydrous potassium carbonate (1.4 g). The reaction mixture was then cooled, filtered, diluted with water (10 mL) and neutralized with 2M hydrochloric acid. The product obtained was filtered, washed with water, dried and recrystallized from acetic acid.

*Monopotassium salt of 3-(4-chlorophenyl)-2-thioxo-2,3,5,6,7,8-hexahydro-1H-[4,5]thieno[2,3-d]-pyrimidin-4-one* (**24)** *and its 2-thioxo derivative* **18a**. Yields 54% (**24**) and 72% (**18a**), recrystallized from ethanol to give a white powder, m.p. > 300 °C; IR (cm^–1^): 3,130 (NH), 3,060, 3,039, 1,693 (CO), 1,524, 1,490 (C=C); ^1^H-NMR (CDCl_3_) δ: 1.85 (4H, m, 2CH_2_), 2.75 (2H, m, CH_2_), 2.91 (2H, m, CH_2_), 7.34 (2H, d, *J* = 8.8 Hz, Ar-CH), 7.55 (2H, d, *J* = 8.8 Hz, Ar-CH), 7.26 (1H, s, NH); MS: *m/z* 348 (M^+^, 50%), 206 (M^+^-C_6_H_5_Cl, SH + 2H, 58%), 111 (M^+^- (C_6_H_5_Cl, SH,2C_2_H_4_, N,+ 3H), 56%). 

*Monopotassium salt of 7-benzyl-3-phenyl-2-thioxo-2,3,5,6,7,8-hexahydro-1H-pyrido[3′,4′:4,5]- thieno[2,3-d]-pyrimidin-4-one* (**25**) *and its 2-thioxo derivative* **18d**. Yields: 49% (**25**) and 59%^A^, 46%^B^ (**18d**); Yellow powder, m.p. 273–274°C (acetic acid); IR (cm^–1^): 3,390 (NH), 3,061, 1,689 (CO), 1,523 (C=C); MS: *m/z* 405 (M^+^, 27%), 314 (M^+^-(CH_2_-Ph), 7%), 286 (M^+^-(CH_2_-Ph, N-CH_2_), 29%). 

### 3.21. 7-Benzyl-3-phenyl-2-hydrazino-5,6,7,8-tetrahydro-1H-pyrido[3′,4′:4,5]thieno[2,3-d]-pyrimidin-4-one (**19**)

A mixture of **18d** (1.62 g, 4 mmol) and 99% hydrazine hydrate (4 mL, 80 mmol) in pyridine (20 mL) was heated under reflux for 15 h. The mixture was evaporated under reduced pressure and the residue was treated with ethanol. The solid product was filtered, washed with ethanol, dried and recrystallized from ethanol [13] to give an orange powder, m.p. 186–187 °C; Yield 75%; IR (cm^–1^): 3,311–3,145 (NH, NH_2_), 3,034, 1,689 (C=O), 1,546, 1,504 (C=C); ^1^H-NMR (CDCl_3_) δ: 2.82 (2H, m, CH_2_), 2.99 (2H, m, CH_2_), 3.61 (2H, s, CH_2_), 3.72 (2H, s, CH_2_), 3.89 (2H, s, NH_2_), 5.40 (s, NH), 7.22 (2H, d, *J* = 8.8 Hz, Ar-CH), 7.38 (2H, d, *J* = 8.8 Hz, Ar-CH), 7.23–7.58 (6H, m, C_6_H_5_); ^13^C-NMR (CDCl_3_) δ: 25.68, 49.97, 51.61, 62.10 (4C, CH_2_), 115.39, 125.48, 133.65, 152.68 (4C, thiophene carbons), [127.36, 128.46 (2C), 128.78 (2C), 129.24 (2C), 129.95, 130.14, 130.69 (2C), 138.14 (12C, 2C6H4)], 158.41 (C=N), 164.85 (C=O); MS: *m/z* 403 (M^+^,47%), 387 (M^+^-(NH_2_), 20%), 312 (M^+^-CH_2_-Ph, 8%), 284 (M^+^-CH_2_-Ph , N-CH_2_, 66%).

### 3.22. General procedure for the preparation of **26–29**

A mixture of thiouredo derivatives **20–23** (10 mmol) and hydrazine hydrate (20 mmol) in ethanol (100 mL) was heated under reflux for 3–4 h. The solid that separated upon cooling was filtered, washed with water, dried and recrystallized from ethanol.

*3-Amino-2-phenylamino-5,6,7,8-tetrahydro-3H-benzo[4,5]thieno[2,3-d]pyrimidin-4-one* (**26**). Fine colorless crystals, m.p. 204–205 °C; Yield 56%; IR (cm^–1^); 3,323, 3,309, 3,259 (NH_2_, NH), 3,034, 1,672 (CO), 1,606, 1,544 (C=C); ^1^H-NMR (DMSO-d_6_) δ: 1.76 (4H, m, 2CH_2_), 2.63 (2H, m, CH_2_), 2.82 (2H, m, CH_2_), 5.59 (2H, s, NH_2_), 7.06 (1H, t, *J* = 7.3 Hz, Ar- CH(*p*)), 7.34 (2H, t, *J* = 7.3 Hz, Ar-CH(*m*)), 7.74 (2H, d, *J* = 8.08 Hz, Ar-CH(*o*)), 9.35 (1H, s, NH); ^13^C-NMR (DMSO-d_6_) δ: 22.42, 23.24, 24.84, 25.77 (aliphatic carbons), 115.19, 127.48, 130.72, 149.30 (thiophene carbons), 121.39, 123.69, 129.14, 138.83 (phenyl carbons), 157.99 (C=N), 166.13 (C=O); MS: *m/z* 312 (M^+^, 20%), 297 (M^+^-NH_2_)+ H, 21%), 235 (M^+^- Ph, 36%), 221 (M^+^-NH Ph + H, 46%). 

*3-Amino-2-(4-chlorophenyl)amino-5,6,7,8-tetrahydro-3H-benzo[4,5]thieno[2,3-d]pyrimidin-4-one* (**27**). colorless crystals, m.p. 166–167 °C; Yield: 46%; IR (cm^–1^); 3,334, 3,330, 3,203 (NH_2_, NH), 3,099, 1,664 (CO), 1,587, 1,546 (C=C); ^1^H-NMR (DMSO-d_6_) δ: 1.78 (4H, m, 2CH_2_), 2.64 (2H, m, CH_2_), 2.87 (2H, m, CH_2_), 4.57 (2H, s, NH_2_), 7.29 (2H, d, *J* = 8.8 Hz, Ar-CH), 7.59 (2H, d, *J* = 8.8 Hz, Ar-CH), 8.53 (s, NH); ^13^C-NMR (DMSO-d_6_) δ: 22.34, 23.11, 25.00, 25.45 (4C, aliphatic ring), 115.54, 128.56, 130.69, 147.15 (4C, thiophene ring); 121.14 (2C), 129.05 (2C), 129.15, 136.41 (aromatic carbons), 158.20 (C=N), 163.09 (C=O); MS: *m/z* 346 (M^+^, 98%), 348 (M^+^+1, 54%), 312 (M^+^-Cl + H, 14%), 330 (M^+^-NH_2_, 15%), 331 (M^+^-NH_2_ + H, 6%), 220 (M^+^-NH_2_, Ph-Cl) +H, 5%), 206 (M^+^-NH_2_, NH Ph-Cl) + 2H, 38%), 179 (M^+^-NH_2_, NH Ph-Cl, HCN + 2H, 32%). 

*7-Benzyl-3-Amino-2-phenylamino-5,6,7,8-tetrahydro-3H-pyrido[3′4′:4,5]thieno[2,3-d]pyrimidin-4-one* (**28**). Light yellow crystals, m.p. 150–152 °C; Yield 32%; IR (cm^–1^): 3,317, 3,300 (NH_2_, NH), 3,032 (Ar-CH), 1,691 (CO), 1,589, 1,543 (C=C); ^1^H-NMR (CDCl_3_) δ: 2.78 (2H, t, CH_2_), 2.99 (2H, t, CH_2_), 3.46 (2H, s, CH_2_), 3.68 (2H, s, CH_2_), 4.81 (2H, s, NH_2_), 7.07 (1H, t, *J* = 7.3 Hz, Ar-CH(*p*)), 7.34 (2H, t, *J* = 8.08 Hz, Ar-CH(m)), 7.55 (2H, d, *J* = 8.08 Hz, Ar-CH(*o*)), 7.40–7.35 (5H, m, C_6_H_5_), 8.42 (1H, s, NH); ^13^C-NMR (CDCl_3_) δ: 25.78, 50.21, 51.60, 62.52 (aliphatic carbons), 114.73, 127.48, 137.73, 147.52 (4C, thiophene carbons), [120.02 (2C), 123.63, 125.61, 128.51, 129.02 (2C), 129.44 (2C), 129.65 (2C), 137.84, aromatic carbons], 157.97 (C=N), 163.66 (C=O); MS : *m/z* 403 (M^+^,49%), 387 (M^+^(-NH_2_ ),14%), 311 (M^+^-(C_6_H_5_, NH),16%), 284 (M^+^-(CH_2_C_6_H_5_, N-CH_2_, 43%), 176(M^+^-(CH_2_C_6_H_5_, N-CH_2_, NH_2_, NH-C_6_H_5_), 9%), 150 (M^+^-(CH_2_C_6_H_5_, N-CH_2_, NH_2_, NH-C_6_H_5_, HCN) + (H), 23%). 

*7-Benzyl-3-Amino-2-(4-chlorophenyl)amino-5,6,7,8-tetrahydro-3H-pyrido[3′4′:4,5]thieno[2,3-d]pyrimidin-4-one* (**29**). Light yellow crystals, m.p. 192–194 °C; Yield: 29%; IR (cm^–1^): 3,450–3,313 (br, NH_2_), 3,201 (NH), 3,032 (Ar-CH), 1,674 (CO), 1,537 (C=C); ^1^H-NMR (CDCl_3_) δ: 2.78 (2H, m, CH_2_), 3.02 (2H, m, CH_2_), 3.44 (2H, s, CH_2_), 3.68 (2H, s, CH_2_), 4.86 (2H, s, NH_2_), 7.25 (2H, d, *J* = 8.8 Hz, Ar-CH), 7.47 (2H, d, *J* = 8.8 Hz, Ar-CH), 7.37–7.30 (5H, m, C_6_H_5_), 8.40 (s, NH); ^13^C-NMR (CDCl_3_) δ: 25.78, 50.31, 51.56, 62.62 (4C, 4CH_2_), 114.88, 127.55, 137.72, 147.25 (4C, thiophene carbons); [121.12 (2C), 125.83, 128.55 (2C), 128.99 (2C), 129.02 (2C), 129.51 (2C), 136.30, 12C, 2C6H5], 157.81 (C=N), 163.35 (C=O); MS : *m/z* 437 (M^+^, 88%), 421 (M^+^ - NH_2_ , 18%), 345 (M^+^-[CH_2_C_6_H_5_] + H, 29%), 318 (M^+^-CH_2_C_6_H_5_,HCN + H, 68%), 302 (M^+^- CH_2_C_6_H_5_, HCN,-NH_2_, +H), 21%), 91 (CH_2_C_6_H_5_, 100%).

### 3.23. Synthesis of **30**, **31**

A mixture of **26** (1.56 g, 5 mmol) and aromatic aldehyde (5 mmol) in acetic acid (30 mL) was heated under reflux for 4 h. Then the mixture was cooled and the solid separated was filtered, dried and recrystallized from a suitable solvent.

*3-[N′-(3,4-Dimethoxybenzylidene)-hydrazino]-2-phenylamino-5,6,7,8-tetrahydro-3H-benzo[4,5]thieno[2,3-d]pyrimidin-4-one* (**30**). Light yellow crystals, m.p. 192–194 °C; Yield 56%; IR (cm^–1^): 3,325 (NH), 3,053, 3,007, 1,672 (CO), 1,600, 1,570 (C=C); MS: *m/z* 460 (M^+^, 24%), 297 (M^+^-(N=CHC_6_H_3_ (OMe)_2_) + H, 44%), 180 (M^+^-(N=CHC_6_H_3_ (OMe)_2_, NHC_6_H_5_, HCN) + 2H, 16%).

*3-[N′-(2,4-Dichlorobenzylidene)-hydrazino]-2-phenylamino-5,6,7,8-tetrahydro-3H-benzo[4,5]thieno[2,3-d]pyrimidin-4-one* (**31**). Yellow powder, m.p. 192–194 °C; Yield 51%; IR (cm^–1^): 3,319 (NH), 3,068, 1,668 (CO), 1,598, 1,537, 1,544 (C=C); MS: *m/z* 468 (M^+^, 57%), 470( M^+^ +2, 33%), 323 (M^+^- (C_6_H_3_Cl_2_), 29% ), 268 (M^+^-(C_6_H_3_Cl_2_,HCN, C_2_H_4_), 96% ), 178 (M^+^-(C_6_H_3_Cl_2_, HCN, C_2_H_4_, NHC_6_H_5_ + 2H, 46% ). 

### 3.24. Synthesis of 4-amino-5,6-disubstituted[4,5]thieno[2,3-d]pyrimidines **33a,b**

A mixture of compound **32a,b** (10 mmol) and formamide (10 mL) was heated under reflux for 2 h, then left to cool overnight at ambient temperature. The solid formed was filtered, dried and recrystallized from ethanol [24].

*7-Benzyl-4-amino-5,6,7,8-tetrahydropyrido[3′,4′:4,5]thieno[2,3-d]pyrimidine* (**33a**). Light brown crystals, m.p. 236–237 °C; yield: 73%; IR (cm^–1^): 3,413, 3,313 (NH_2_), 3,050, 1,633, 1,552 (C=C); ^1^H-NMR (DMSO-d_6_) δ: 2.77 (2H, t, CH_2_), 2.96 (2H, t, CH_2_), 3.62 (2H, s, CH_2_), 3.70 (2H, s, CH_2_), 6.85 (2H, s, NH_2_), 7.27–7.36 (5H, m, C_6_H_5_), 8.18 (1H, s, H-2); ^13^C-NMR (DMSO-d_6_) δ: 26.17, 49.43, 51.93, 61.16 (4C, 4CH_2_), 115.02, 127.68, 138.71, 153.70 (thiophene carbons), 126.13, 128.85 (2C), 129.13, 129.37, (2C) (C_6_H_5_), 158.72 (C-2), 166.30 (C-4); MS : *m/z* 296 (M^+^, 25%), 205 (M^+^-(CH_2_-C_6_H_5_), 50%), 177 (M^+^-(CH_2_-C_6_H_5_,NCH_2_), 63%), 162 (M^+^- ( CH_2_-C_6_H_5_,NCH_2_, NH_2_ ) + H, 2%).

*4-Amino-6,7,8,9-tetrahydro-5H-cyclohepta[4,5]thieno[2,3-d]pyrimidine* (**33b**). Violet crystals, m.p. 264–266 °C; Yield: 66%; IR (cm^–1^): 3,352, 3,327 (NH_2_), 3,143, 1,651, 1,556 (C=C); ^1^H-NMR (DMSO-d_6_) δ: 1.65 (4H, m, 2CH_2_), 1.83 (2H, m, CH_2_) 2.83 (2H, t, CH_2_), 3.00 (2H, t, CH_2_), 6.92 (2H, s, NH_2_), 8.15 (1H, s, H-2); ^13^C-NMR (DMSO-d_6_) δ: 26.98, 27.40, 29.11 (2C), 31.34 (5C, 5 CH_2_), 116.66, 132.68, 135.55, 152.96 (4C, thiophene carbons), 158.70 (C-2), 161.79 (C-4); MS: *m/z* 219 (M^+^, 100%), 204 (M^+^-(NH_2_) + H, 23%), 190 (M^+^-(NH_2_, CH_2_) + H, 52%). 

### 3.25. (3-Cyano-5,6,7,8-tetrahydro-4H-cyclohepta[b]thiophene-2-yl)-carbamic acid ethyl ester (**34**) 

A mixture of **32b** (3.84 g, 20 mmol) and ethyl chloroformate (2.3 mL, 24 mmol) in pyridine (40 mL), was stirred for 30 h at room temperature, the solvent was removed under reduced pressure to obtain a dark oil, treated with 3–5 mL of water, the the precipitate collected by filtration, dried and recrystallized from ethanol to give violet crystals of **34**, m.p. 135–137 °C; Yield: 49%; IR (cm^–1^): 3,215 (NH), 2,223 (CN), 1,724 (CO), 1,571, 1,556 (C=C), 1,240 (C-O); ^1^H-NMR (CDCl_3_) δ: 1.33 (3H, t, *J* = 7.32 Hz, CH_3_), 1.64 (4H, m, 2CH_2_), 1.82 (2H, m, CH_2_), 2.67 (4H, m, 2CH_2_), 4.26 (2H, q, *J* = 7.32 Hz, CH_2_-O), 7.69 (1H, s, NH); ^13^C-NMR (CDCl_3_) δ: 14.44 (CH_3_), 27.34, 28.06, 29.13, 29.22, 32.04 (5C, 5CH_2_), 62.87 (CH_2_-O), 95.30, 130.98, 136.04, 146.13 (4C, thiophene carbons), 114.81 (CN), 152.67 (C=O). 

### 3.26. 2,3,5,7,8,9,10,11-Octahydro-[1,3]-imidazolo[2,3-c]-4-oxo-cyclohepta[4,5]thieno[2,3-d]pyrimidine (35)

Ethylenediamine (1 mL) was added dropwise with stirring to a solution of compound **34** (264 mg, 1 mmol) in DMF (5 mL) within 2 h at 150 °C. After cooling, water (20 mL) was added to the solution and the precipitated crystals were collected by filtration, washed with water, dried and crystallized from ethanol to give colorless crystals, m.p. 275–276 °C; yield: 80%; IR (cm^–1^): 3,138 (NH), 1,643 (CO), 1,573, 1,525 (C=C); ^1^H-NMR (CDCl_3_) δ: 1.52 (4H, m, 2CH_2_), 1.69 (2H, m, CH_2_), 2.53 (2H, t, *J* = 5.8 Hz, CH_2_), 2.94 (2H, t, *J* = 5.8 Hz, CH_2_), 3.79 (2H, m, N-CH_2_), 3.90 (2H, m, N-CH_2_), 2.44 (1H, s, NH); ^13^C-NMR (CDCl_3_) δ: 24.17, 24.85, 25.10, 26.31, 29.09 (5C, 5CH_2_), 40.04 (2C, 2CH_2_-N), 112.00, 121.50, 127.35, 132.11 (thiophene carbons), 146.21 (CN), 149.52 (C=O); MS: *m/z* 261 (M^+^, 100%), 220 (M^+^-(NCH_2_CH_2_) + H, 50%).

### 3.27. 7-Benzyl-4-imino-3-amino-5,6,7,8-tetrahydro-4H-pyrido[3′,4′:4,5] thieno [2,3-d] pyrimidine (**36**)

A mixture of **32a** (20 mmol) and triethylorthoformate (20 mL) was heated under reflux for 4 h, and the excess of the reagent was then removed under vacuum, A mixture of hydrazine hydrate (99%, 5 mL) and ethanol (15 mL) was added to the residue with stirring, and it was allowed to stand overnight at room temperature. The solid obtained was filtered, washed with ethanol, dried and crystallized from ethanol. Fine brown crystals, m.p. 166–167 °C; Yield: 67%; IR (cm^–1^); 3,354, 3,253, 3,174 (NH_2_, NH), 3,094–3,031 (Ar-CH), 1,652, 1,606 (C=N), 1,554, 1,541 (C=C); ^1^H-NMR (CDCl_3_) δ: 2.98 (2H,t,CH_2_), 3.21 (2H, s, CH_2_), 3.72 (4H, m, 2 CH_2_), 5.42 (2H, s, NH_2_), 7.25-7.37 (5H, m, C_6_H_5_), 8.08 (H, s, CH), 8.87 (1H, s, NH).

*7-Benzyl-4-imino-3-amino-2-methyl-5,6,7,8-tetrahydro-4H-pyrido[3′,4′:4,5]* *thieno[2,3-d]* *pyrimidine* (**37**). Prepared from **32a** (20 mmol) and triethyl orthoacetate (20 mL) following the same procedure as for **36** above. Fine light brown crystals, m.p. 172–174 °C; Yield : 54%; IR (cm^–1^): 3,330, 3,284, 3,178 (NH_2_, NH), 3,094–3,028 (Ar-CH), 1,652, 1,610 (C=N), 1,544, 1,521 (C=C); ^1^H-NMR (CDCl_3_) δ: 2.79 (3H, s, CH_3_), 2.83 (2H, t, *J* = 5.8 Hz, CH_2_), 2.97 (2H, t, *J* = 5.8 Hz, CH_2_), 3.58 (2H, s, CH_2_), 3.69 (2H, s, CH_2_), 6.14 (2H, br, s, NH_2_), 7.34–7.28 (6H, m, NH, C_6_H_5_); ^13^C-NMR (CDCl_3_) δ: 22.75 (CH_3_), 26.18, 49.43, 51.70, 61.94 (4C, 4CH_2_), 115.59, 127.18, 137.53, 154.59 (thiophene carbons), 127.50, 128.57 (2C), 128.18 (2C), 133.00 (6C, C_6_H_5_), 156.05 (C-2), 160.50 (C-4).

*4-Imino-3-amino-6,7,8,9-tetrahydro-4H,5H-cyclohepta[4,5]thieno[2,3-d]pyrimidine* (**38**). Prepared from **32b** (20 mmol) with triethyl orthoformate (20 mL) following the procedure above described for **36**. Fine colorless crystals, m.p: 164–165 °C; yield: 56%; IR (cm^–1^): 3,294, 3,286, 3,163 (NH_2_, NH), 3,053 (Ar-CH), 1,639, 1,614 (C=N), 1,562 (C=C); ^1^H NMR (CDCl_3_) δ: 1.79 (4H, m, 2CH_2_), 1.91 (2H, m, CH_2_), 2.74 (2H, m, CH_2_), 3.06 (2H, m, CH_2_), 4.75 (2H, brs, NH_2_), NH not observed, 7.96 (1H, s, C^2^-H); ^13^C-NMR (CDCl_3_) δ: 25.53, 27.34, 29.23, 29.36, 31.20 (5C, 5 CH_2_), 120.44, 135.02, 138.08, 146.41 (4C, thiophene carbons), 155.31 (C-2), 155.99 (C-4).

4-Imino-3-amino-2-methyl-6,7,8,9-tetrahydro-4H,5H-cyclohepta[4,5]thieno*[2,3-d]* pyrimidine (39). Prepared from 32b (20 mmol) with triethyl orthoacetate (20 mL) following the procedure of 36. Fine colorless crystals, m.p. 186–188 °C; Yield: 70%; IR (cm^–1^); 3,354, 3,284, 3,190 (NH_2_, NH), 3,001 (Ar-CH), 1,647, 1,606 (C=N), 1,552, 1,521 (C=C); ^1^H-NMR (CDCl_3_) δ: 1.71 (4H, m, 2CH_2_), 1.83 (2H, m, CH_2_), 2.67 (3H, s, CH_3_), 2.77 (2H, m, CH_2_), 3.00 (2H, m, CH_2_), 5.50 (2H, br, s, NH_2_), NH not observed; ^13^C-NMR (CDCl_3_) δ: 22.53 (1C, CH_3_), 26.41, 27.21, 29.14, 29.25, 30.96 (5C, 5 CH_2_), 117.51, 134.13, 138.23, 154.94 (thiophene ring), 155.55 (C-2), 157.28 (C-4). 

### 3.28. 8-Benzyl-2-methyl-7,8,9,10-tetrahydropyrido[4′,3′-4,5]thieno[3,2-e][1,2,4]triazolo[2,3-c]pyrimidine (**40**)

Compound **36** (10 mmol) was heated under reflux for 2 h with an excess of triethyl orthoacetate(20 mL) and the excess of the reagent was then removed under vacuum. The solid obtained was washed with ethanol, dried and crystallized from ethanol to give brown crystals, m.p. 157–159 °C; Yield: 45%; IR (cm^–1^): 3,041 (Ar-CH), 1,618 (C=N), 1,556, 1,508 (C=C); ^1^H-NMR (CDCl_3_) δ: 2.65 (3H, s, CH_3_), 2.99 (2H, m, CH_2_), 3.27 (2H, m, CH_2_), 3.79 (4H, s, 2CH_2_), 7.34–7.38 (5H, m, C_6_H_5_), 9.06 (1H, s, CH); ^13^C-NMR (CDCl_3_) δ: 14.78 (CH_3_), 25.62, 49.79, 51.77, 61.91 (4C, 4 CH_2_), 119.65, 127.43, 136.20, 137.79 (thiophene carbons), 127.52, 128.55 (2C), 129.12 (2C), 135.44 (6C, C_6_H_5_), 149.29 (C=N), 154.06 (C=N), 165.25 (C=N); MS : *m/z* 335 (M^+^, 11%), 244 (M^+^-(CH_2_-C_6_H_5_), 10%), 216 (M^+^-(CH_2_-C_6_H_5_, NCH_2_), 37%).

*8-Benzyl-4-methyl-7,8,9,10-tetrahydro-pyrido[4′,3′-4,5]thieno[3,2-e][1,2,4]triazolo[2,3-c]pyrimidine* (**41**). Prepared from **37** (10 mmol) and triethyl orthoformate (20 mL) following the procedure given for **40**. Fine light yellow scales, m.p. 142–144 °C; Yield 39%; IR (cm^–1^); 3,084–3,060 (Ar-CH), 1,652, 1,622 (C=N), 1,558, 1,517 (C=C); ^1^H-NMR (DMSO-d_6_) δ: 2.97 (3H, s, CH_3_), 3.04 (2H, m, CH_2_), 3.30 (2H, br, s, CH_2_), 3.82 (4H, m, 2CH_2_), 7.26-7.40 (5H, m, C_6_H_5_), 8.36 (1H,s,CH); MS: *m/z* 335 (M^+^, 8%), 244 (M^+^-(CH_2_-C_6_H_5_), 30%), 216(M^+^-(CH_2_-C_6_H_5_, NCH_2_), 100%).

*4-Methyl-8,9,10,11-tetrahydro-7H-cyclohepta[4,5]thieno[3,2-e][1,2,4]triazolo[2,3-c]pyrimidine* (**42**). Prepared from **39** (10 mmol) and triethyl orthoformate (20 mL) following the procedure given above for **40**. Fine colorless crystals, m.p. 159–160 °C; Yield: 67%; IR (cm^–1^): 3,089 (Ar-CH), 1,618 (C=N), 1,550, 1,521 (C=C); ^1^H-NMR (CDCl_3_) δ: 1.74 (4H, m, 2 CH_2_), 1.92 (2H, m, CH_2_), 2.91 (2H, m, CH_2_), 2.95 (3H, s, CH_3_), 3,41 (2H, m, CH_2_), 8.34 (1H, s, C-H); ^13^C-NMR (CDCl_3_) δ: 19.64 (CH_3_), 27.34, 27.85, 28.41, 30.46,32.42 (5C, 5CH_2_), 120.55, 134.11, 141.56, 145.43 (4C, thiophene carbons), 149.04 (C=N), 152.01 (C=N), 153.32 (C=N); MS: *m/z* 258 (M^+^, 100%), 243 (M^+^-(CH_3_), 37%), 230 (M^+^-(C_2_H_4_), 69%), 216 (M^+^-(C_2_H_4_ , CH_3_) + H, 18%).

*4,2-Dimethyl-8,9,10,11-tetrahydro-7H-cyclohepta[4,5]thieno[3,2-e][1,2,4]triazolo[2,3-c]pyrimidine* **(43)**. Prepared from **39** (10 mmol) and triethyl orthoacetate (20 mL) following the procedure described above for **40**, Fine colorless crystals, m.p. 189–190 °C; Yield: 64%; IR (cm^–1^): 2,922–2,854 (aliphatic CH), 1,618 (C=N), 1,550, 1,519 (C=C). ^1^H-NMR (CDCl_3_) δ: 1.77 (4H, m, 2 CH_2_), 1.93 (2H, m, CH_2_), 2.64 (3H, s, CH_3_), 2.94 (5H, CH_2,_ CH_3_), 3,42 (2H, m, CH_2_). ^13^C-NMR (CDCl_3_) δ: 14.63 (CH_3_), 19.64 (CH_3_), 27.3, 27.8, 28.37, 30.4, 32.1 (5C, 5CH_2_), 119.89, 133.89, 140.71, 151.96 (thiophene carbons), 149.51 (C-4), 144.88 (C-10), 163.47 (C-2); MS: *m/z* 272 (M^+^, 82%), 243 (M^+^-(2CH_3_)+H, 60%), 230(M^+^-(CH_3_,C_2_H_4_)+H, 47%).

### 3.29. 8-Benzyl-7-8,9,10-tetrahydro-3H-pyrido[4′,3′:4,5]thieno[3,2-e][1,2,4]triazolo[2,3-c]pyrimidine-2-thione (**44**) 

A mixture of **36** (1 mmol) in pyridine (10 mL) and carbon disulfide (400 mg, 5 mmol) was heated under reflux for 3 h. The solid obtained after cooling, was collected and recrystallized from ethanol. Fine orange scales, m.p. 237–238 °C; Yield: 36%; IR (cm^–1^): 3,379 (NH) 3,028 (Ar-CH), 2,850–2,920 (aliphatic CH), 1,525, 1,454 (C=C), 1,392 (C=S); ^1^H-NMR (CDCl_3_) δ:2.87 (2H, m, CH_2_), 3.68 (2H, m, CH_2_), 4.01 (4H, m, 2CH_2_), 7.50–7.52 (3H, m), 7.41–7.68 (2H, m), 8.14 (1H, s, H-2). 

### 3.30. 8,9,10,11-Tetrahydro-3H,7H-cyclohepta[4,5]thieno[3,2-e][1,2,4]triazolo[2,3-c]pyrimidine-2-thione (**45**)

A mixture of **38** (1 mmol) in pyridine (10 mL) and carbon disulfide (400 mg, 5 mmol) was heated under reflux for 3 h. Work up following the procedure given above for **44** and recrystallization from ethanol gave **45** as yellow scales, m.p. 275–276 °C; Yield: 75%; IR (cm^–1^): 3,057 (Ar-CH), 2,9845–2,999 (aliphatic CH), 1,517, 1,454 (C=C), 1,365 (C=S). ^1^H-NMR (CDCl_3_) δ: 1.70–2.0 (6H, m, 3 CH_2_), 2.98 (4H, m, 2 CH_2_), 9.43 (1H, s, NH); ^13^C-NMR (CDCl_3_) δ: 26.56, 26.96, 28.99, 30.65, 31.38 (5C, 5CH_2_), 125.21, 132.25, 149.53, 153.5 (4C, thiophene carbons), 135.65 (C-2), 157.67 (C-4), 182.39 (C=S).

*4-Methyl-8,9,10,11-tetrahydro-3H,7H-cyclohepta[4,5]thieno[3,2-e][1,2,4]triazolo[2,3-c]pyrimidine-2-thione* (**46**). Prepared from **38** (1 mmol) in pyridine (10 mL) and carbon disulfide (400 mg, 5 mmol) following procedure described for **45**. Yellow scales, m.p. 288–290 °C; Yield: 80%; IR (cm^–1^): 2,916, 2,846 (aliphatic CH_2_), 1,618 (C=N), 1,550, 1,519 (C=C); ^1^H-NMR (CDCl_3_) δ: 1.77 (4H, m, 2 CH_2_), 1.93 (2H, m, CH_2_), 2.91 (2H, m, CH_2_), 2.98 (2H, m, CH_2_), 3.03 (3H, s, CH_3_); ^13^C-NMR (CDCl_3_) δ: 22.37 (1C, CH_3_), 26.52, 26.96, 28.57, 30.35, 31.24 (5C, 5 CH_2_), 123.90, 131.68, 146.95, 153.68 (4C, thiophene carbons), 153.68 (C-2), 157.94 (C-4), 181.21 (C=S).

## 4. Conclusions

Several new thienopyrimidin-4-one(thione) derivatives were synthesized from 2-amino-3-carbethoxy (or 3-cyano)-4,5-disubstituted thiophenes as starting materials. Further, some triazolo- thienopyrimidine and 2-thioxothienopyrimidine representatives have also been synthesized, and their structures have been determined on the basis of their 2D NMR data. Some of prepared compounds were evaluated for their antimicrobial and antitumor -activities, and compounds **4a**, **12**, **15** were found to be promising antimicrobial agents, while compounds **7a**, **8a**, **15**, **29**, **42** have some activity against different human tumor cell lines.

## Figures and Tables

**Table 1:**
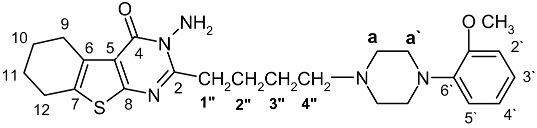
^1^H-NMR, ^13^C-NMR and HMBC correlations of **4a**(CDCl_3_).

No	^13^-NMR(DEPT)	^1^H-NMR	HMBC	
	δ_C_	δ_H_	^2^*J*	^3^*J*
1	-			
2	156.97 (C)			
3	-	4.96 (NH_2_)		C-2
4	161.57 (C)			
5	158.18 (C)			
6	130.91 (C)			
7	119.87 (C)			
8	141.34 (C)			
9	25.16 (CH_2_)	2.71	C-6, 10	C-5, 7, 11
10	22.24 (CH_2_)	1.83	C-9, 11	C-6, 12
11	22.95 (CH_2_)	1.83	C-10, 12	C-7, 9
12	25.39 (CH_2_)	2.95	C-7, 11	C-6, 10
1′	152.29 (C)			
2′	111.31 (CH)	6.83(d)*	C-1′, 3′	C-4′, 6′
3′	121.01 (CH)	6.88-6.93	C-2′, 4′	C-1′, 5′
4′	122.84 (CH)	6.97(t)*	C-3′, 5′	C-2′, 6′
5′	118.22 (CH)	6.88-6.93	C-4′, 6′	C-1′, 3′
6′	133.05 (C)			
O-CH_3_	55.34 (CH_3_)	3.84		C-1′
**1ʺ**	33.99(CH_2_)	2.98(t)	C-**2ʺ,** 2	C-**3ʺ**
**2ʺ**	26.25(CH_2_)	1.65	C-**1ʺ**, **3ʺ**	C-2, **4ʺ**
**3ʺ**	24.65(CH_2_)	1.83	C-**2ʺ**, **4ʺ**	C-**1ʺ**
**4ʺ**	58.32(CH_2_)	2.47(t)	C-**3ʺ**	C-**a**, **2ʺ**
**a**	53.75(2CH_2_)	2.66(2 CH_2_)	C-**a′**	C-**4ʺ**
**a′**	50.33(2CH_2_)	3.09(2 CH_2_)	C-**a**	C-6′

* *J* = 7.0 Hz.

**Table 2:** Antimicrobial activity of the select synthesized compounds.

	Diameter of inhibition zone (mm)			
	Gram-positive		Gram -negative	
	*S.a*	*K.m*	*P.a*	*E.c*
4a ^**^	12	4	6	13
8c ^**^	16	0	0	4
12 ^*^	12	4	0	0
15 ^*^	11	7	4	6
17 ^*^	0	0	4	0
41 ^*^	9	2	1	0
46 ^**^	9	3	7	2
**CAZ**	-	9	28	20
**VA**	17	-	-	-

_*_ DMF; _**_ CHCl_3._

**Table 3:** Results of antitumor cytotoxicity for some synthesized thienopyrimidines.

Comp. No	Cytotoxicity IC_50_ (μg/mL)				
	HEPG2	HELA	MCF7	HCT116	HEP2
**4a**	2.35	1.07	2.82	2.01	1.61
**7d**	1.28	1.41	0.67	0.47	0.47
**8a**	0.94	0.87	0.74	-	0.47
**12**	0.74	0.81	1.54	2.68	0.81
**15**	1.21	0.40	0.6	1.68	0.54
**29**	0.40	0.60	0.94	2.89	1.14
**42**	1.81	0.81	2.48	0.54	1.14
**Doxorubicin**	0.54	0.85	0.7	0.69	0.4
